# Maternal supplementation of vitamin B12 in predominantly vegetarian pregnant women improves their vitamin B12 status and the neurodevelopment of their infants: the MATCOBIND multicentric double-blind randomised control trial

**DOI:** 10.1136/bmjpo-2025-004112

**Published:** 2026-03-18

**Authors:** Jitender Nagpal, Manu Mathur, Swapnil Rawat, Atul Singhal, Rajendra Pant, Anita Shah, Laura Nixon, Vijay Kumar Mishra, Deepti Nagrath, Michelle Heys, Mario Cortina-Borja, Colin Michie, Jageshwor Gautam, Shailendra Bir Karmacharya, Snighda Rai, Monica Lakhanpaul

**Affiliations:** 1 Department of Pediatrics & Clinical Epidemiology, Sitaram Bhartia Institute of Science and Research, New Delhi, Delhi, India; 2 Queen Mary University of London Faculty of Medicine and Dentistry, London, UK; 3 Public Health Foundation of India, New Delhi, India; 4 Department of Clinical Epidemiology, Sitaram Bhartia Institute of Science and Research, New Delhi, Delhi, India; 5 UCL Great Ormond Street Institute of Child Health, London, UK; 6 Paropakar Maternity and Women’s Hospital, Kathmandu, Nepal; 7 Wolfson Institute of Population Health, UCL, London, UK; 8 University College London, London, UK; 9 Department of Neonatology, Paropakar Maternity and Women's Hospital, Kathmandu, Nepal; 10 Nottingham University NHS Trust, Nottingham, UK

**Keywords:** Infant, Low and Middle Income Countries, Mothers, Child Health

## Abstract

**Background:**

Vitamin B_12_ deficiency is common in populations with limited animal-source foods and has been linked to delayed infant neurodevelopment and adverse pregnancy outcomes. Evidence on the benefits of maternal B_12_ supplementation for improving infant neurodevelopment remains mixed, particularly in low-income and middle-income countries where deficiency is prevalent.

**Methods:**

This double-blind, randomised controlled trial was conducted in two tertiary maternity care centres in India and Nepal. Pregnant women in their first trimester, following a vegetarian diet, were enrolled and randomised to receive either a daily oral supplement of 250 µg (group A) or 50 µg (group B) of methyl-cobalamin from enrolment to 6 months post partum. The primary outcomes were infant neurodevelopment assessed at 9–12 months using the Development Assessment Scale of Indian Infants and biochemical B_12_ status in mothers and infants measured through blood tests.

**Results:**

531 mothers completed the study (group A n=255; group B n=276). There were no significant differences in baseline characteristics between mothers at both centres or in groups A and B. Mental developmental quotients (DQs) were higher in the infants of group A: 103.7 (7.7) than group B: 101.7 (8.8); p=0.008). This corresponds to a mean difference of 7.8 centiles (p=0.007). Mean motor DQs were not significantly different between the groups. Maternal vitamin B_12_ levels rose from the first to third trimester in both groups, with a larger increase in group A (104.9 pg/mL (SD 159.1)) than group B (47.5 pg/mL (SD 118.0))*,* p<0.001. Holotranscobalamin levels improved similarly (p<0.001). All infant levels of vitamin B_12_ were within the normal range. Newborn anthropometry, APGAR scores and morbidity profiles were similar in both groups (p>0.05). Serum ferritin, vitamin D and folate were similar (p>0.05).

**Conclusions:**

Daily supplementation with 250 µg of vitamin B_12_ during pregnancy in vegetarian mothers significantly improved infant mental DQ and maternal B_12_ status compared with a 50 µg dose.

**WHAT IS ALREADY KNOWN ON THIS TOPIC**

- Adequate vitamin B_12_ before and during pregnancy is crucial for maternal metabolism and healthy fetal/newborn brain and nervous system development.

**WHAT THIS STUDY ADDS**

- In vegetarian pregnant women, 250 µg/day B_12_ supplementation led to a meaningful improvement in infant neurodevelopment (~2 developmental quotient points) compared with 50 µg/day. Higher-dose supplementation (250 µg/day) also improved maternal biochemical B_12_ status during pregnancy.

**HOW THIS STUDY MIGHT AFFECT RESEARCH, PRACTICE OR POLICY**

- Strengthens the case for dose–response trials of B_12_ in pregnancy (especially in vegetarian populations), with longer-term child neurodevelopment follow-up.
- Supports routine antenatal B_12_ supplementation for vegetarian women and helps inform maternal nutrition guidelines in settings where low animal-source diets make deficiency more likely.

## Introduction

 Micronutrient deficiencies in pregnancy can be associated with poor infant neurocognition that in turn contributes to suboptimal educational achievement and ultimately economic productivity.^1 2^ Observational studies report that biochemical evidence of deficiency of vitamin B_12_ during pregnancy or early infancy is associated with subsequent delays in measures of infant neurodevelopment,^3^ including cognitive functions,^4 5^ memory,^6^ language and motor skills.^7^ B_12_ deficiency has also been associated with intrauterine growth restriction, preterm delivery and neural tube defects.^8^,^10^ Biochemical measures have shown that vitamin B_12_ status tends to fall from preconception levels across the time period of the pregnancy. Vegetarian diets typically supply very little B_12_ to mothers, their milk and therefore to their infants.^11 12^ Vitamin B_12_ deficiency has been found to be common in many low-income and middle-income countries (LMICs), although its exact prevalence is dependent on the biochemical cut-offs used.^13^,^15^

Evidence from infant vitamin B_12_ supplementation trials is mixed. In a large community-based trial in Nepal, daily oral vitamin B_12_ supplementation did not improve Bayley-III neurodevelopmental scores.^16^ However, some randomised trials in high-income settings (eg, Norway), particularly among infants with biochemical evidence of impaired cobalamin function, have reported improvements in motor function and related clinical symptoms after cobalamin supplementation.^7 17^ Similarly, maternal supplementation trials have also delivered mixed outcomes.^18^,^20^ In a study by Srinivasan *et al*, maternal vitamin B_12_ supplementation with 50 µg daily, from <14 weeks gestation through to 6 weeks post partum, did not improve maternal B_12_ levels in the supplemented group and there was deterioration in B_12_ levels in a placebo cohort.^18^ Infant neurodevelopmental outcomes at 9 months were comparable between the two groups, but a marker of maternal B_12_ insufficiency during pregnancy, hyperhomocysteinaemia, was negatively associated with infant cognitive outcomes. Longer-term follow-up from this same study at 30 months reported an improvement in expressive language in those infants whose mothers were supplemented with vitamin B_12_.^19^

A trial in South India using long-term supplementation with 2 µg/day vitamin B_12_ in severely deficient women aimed to improve their status preconception, also documented improvement in the cognitive and language domains of a BSID-III (Bayley's Scales of Infant Development - III) assessment of their infants.^20^ By contrast, a recent trial from Nepal compared a maternal supplement of 50 µg/day versus placebo and found no difference in infant neurodevelopmental scores at 6 and 12 months, although there was biochemical improvement in maternal cobalamin status with the supplement.^21^ Therefore, the evidence of the efficacy of vitamin B_12_ supplementation during pregnancy, when using early infant neurodevelopment as an outcome, is weak and pointing in both directions.

Hence, we undertook this trial named MATCOBIND to assess the efficacy of vitamin B_12_ supplementation in pregnant women, primarily those following a vegetarian diet, with respect to improving maternal B_12_ concentrations and infant neurodevelopmental outcomes. The research was carried out at two major maternity care centres located in India and Nepal, both of which routinely provide antenatal care that includes iron and folic acid supplements (India: 100 mg iron/500 µg folic acid; Nepal: 60 mg iron/400 µg folic acid). Participants were selected chiefly from the vegetarian population due to their elevated risk of vitamin B_12_ deficiency^22 23^ and potential benefit from supplementation.^24^ The study’s design specifically sought to address limitations observed in previous trials, such as insufficient dosing or abbreviated duration—often ceasing at delivery—which may have contributed to unchanged cognitive results in infants due to restricted exposure. Hence, we decide to compare two daily oral doses of methylcobalamin: 250 µg (group A) and 50 µg (group B) starting from first trimester up to 6 months post partum with rigorous biochemical monitoring of mother–infant B_12_ status to confirm biological separation between groups. The higher dose, 250 µg, was chosen based on earlier evidence of its ability to improve maternal B_12_ levels during pregnancy. We did not use a true placebo, as prior research has shown that withholding B12 can cause maternal levels to decline,^18^ raising ethical concerns. Instead, the 50 µg/day group served as a lower-dose comparator to prevent deficiency rather than act as a biological ‘null’. This dosage has previously been shown to maintain maternal B_12_ status in pregnant women in India.^24^ We, therefore, compared 250 µg/day with 50 µg/day methylcobalamin in predominantly vegetarian pregnant women to assess effects on maternal B_12_ status and infant neurodevelopment at 9–12 months.

## Methods

The study methodology has been documented in detail in previously published protocol paper.^25^ Accordingly, a concise summary is provided here.

### Study design

This multicentre double-blind randomised controlled trial was conducted between October 2018 and September 2022 in two study centres:

- SBISR, New Delhi, India, is a privately funded, pay for care tertiary hospital caring for mothers from a high-income and middle-income population, with approximately 700 deliveries annually (50%–60% mothers were vegetarian, based on preproposal feasibility work)
- The Paropakar Maternity and Women’s Hospital (PMWH), Kathmandu, Nepal is a government hospital caring for mothers from a lower socioeconomic spectrum with approximately 22 000 deliveries annually (5%–10% mothers were vegetarian based on preproposal feasibility work).

#### Participants

Potentially eligible women were screened on their presentation to the antenatal clinics at <12 weeks of gestation between April 2019 and April 2021 to recruit 360 subjects from each study site (n=720).

Inclusion criteria:

1. Consuming a vegetarian diet—including veganism and/or people who do eat egg and/or do consume milk and/or a portion of meat/chicken/fish<once a month.
2. Residing in Delhi NCR or within 10 km of PMWH in Kathmandu, Nepal.
3. Able to understand English, Hindi or Nepalese language.

Exclusion criteria:

1. Intention for medical pregnancy termination.
2. Age <18 or >35 years (later modified to 40, see supplement).
3. Use of vitamin B_12_ supplements.
4. Multiple gestation.
5. Chronic medical conditions (eg, diabetes, hypertension, heart, neurological, thyroid disease).
6. Positive for hepatitis B, HIV or syphilis.
7. Anticipated move out of study area.
8. Infertility treatment.
9. Prediagnosed mental health disorder (depression, drug/alcohol abuse).
10. Recent/current participation in another study (within 4 weeks before trial start).
11. Allergy to vitamin B_12_ or supplement components.

Trial’s exit criteria:

1. Major congenital malformation diagnosis.
2. Infant APGAR (Appearance Pulse Grimace Activity & Respiration) score under 7 at 5 min.
3. Extreme prematurity (less than 28 weeks).
4. Severe growth restriction (SGA (Small for Gestational Age) below third centile by WHO).^26^
5. Neonatal seizures.
6. Healthcare provider request.

### Patient and public involvement

A panel was constituted of eight parents (four couples, with two couples from each site, all of whom had delivered a baby in the past 2 years). This panel reviewed and helped refine participant-facing materials (eg, information leaflets), the consent and recruitment processes, and follow-up plans, ensuring they were clear and acceptable. One parent pair from the panel continued to serve on the trial oversight committee to provide ongoing lay perspectives. Additionally, we conducted exploratory qualitative interviews with pregnant women at both trial sites to identify likely facilitators and barriers to participation. These patient and public involvement (PPI) activities revealed that participants had low awareness of vitamin B_12_ sources and its importance but were keen to learn more, were generally willing to participate in the intervention (including taking supplements and allowing additional infant blood samples), and voiced concerns about potential supplement side effects, mid-trial relocation and out-of-pocket costs. Insights from this PPI work led to concrete protocol modifications: we developed vitamin B_12_ educational materials (posters and community workshops) to address knowledge gaps, adjusted the consent approach so that the routine antenatal clinician introduces the study before detailed consent is obtained by research staff, implemented weekly reminder text messages and arranged reimbursement of travel and test expenses to improve adherence and retention, and tailored our study instruments. By incorporating a home environment assessment tool (Bradley’s HOME inventory)^27^ to capture site-specific context and culturally adapting a prevalidated vitamin B_12_ food frequency questionnaire (FFQ) to reflect local dietary habits. These participant-informed measures ensured that the trial’s design and materials were optimised for feasibility and relevance to the target population.

### Definition of predominantly vegetarian

The study includes vegans and mothers who reported consuming eggs, milk or meat (including chicken or fish) less than once per month. This criterion was based on pilot work at the Nepal site, which indicated that even among non-vegetarian households, more expensive non-vegetarian foods were generally eaten only on festive or celebratory days.

### Randomisation and masking

Eligible and consenting participants were randomised at enrolment to group A (to receive a supplement of 250 µg vitamin B_12_ daily), or group B (to receive a supplement of 50 µg vitamin B_12_ daily). The randomisation sequence was generated at a third-party location (non-study site) using a computer-generated series stratified for location. The sequence was allocated to serial numbers using sequentially numbered, sealed, opaque envelopes. Codes within these envelopes identified boxes labelled with five-digit codes. Each box contained sufficient doses to cater to the study period of one participant. The strips used for both intervention and control B_12_ doses were produced to be indistinguishable in taste, smell and appearance (CADILA Pharma, India; Nova Genetica, Nepal; both manufacturers are GMP accredited, and the products were quality tested for potency and stability). All participants, recruiters, developmental therapists, laboratory personnel, research staff and the data analyst remained blinded to the B_12_ allocation until the final analysis results were disclosed.

### Study procedures

#### Enrolment

Maternal demographic and clinical details were collected from eligible participants, including age, height, weight, ethnicity, education and socioeconomic status. Participants were allocated a study identification number, and their preferred channel of subsequent communication (phone, text message, email) was recorded. A digital weighing scale (accurate to 0.1 kg) and a stadiometer (accurate to 0.1 cm) were used to measure mothers’ weight and height while wearing summer clothing. Participants were fully informed of the nature of the supplement and its administration by the research staff before being given B_12_ supplement doses to last until 5 days beyond the expected date of their next visit. To maximise compliance, weekly supplement and preappointment reminders were sent using the prespecified preferred mode of communication. Empty supplement packets were collected at each appointment as a further indicator of adherence. Maternal tolerance of the B_12_ supplements, including any gastrointestinal symptoms, was recorded at each visit, as was maternal compliance. Supplements were stopped for all mothers at 6 months post partum.

#### Maternal follow-up

Maternal visits took place monthly with their obstetrician until 36 weeks and weekly thereafter as part of routine care. Any acquired morbidities (gestational diabetes, hypertension and hypothyroidism) and medication history were noted in case record forms. In the third trimester, mothers’ dietary B_12_ intake was assessed using a prestandardised and adapted FFQ, and blood sampling was repeated using the same procedures as in the first trimester.

Maternal blood samples were collected at enrolment and in the third trimester. A 12 mL of blood was drawn from each participant into a serum separator vial and an EDTA tube.

#### Childbirth

A medical officer and/or paediatrician monitored the birth and post-delivery course of newborns. Gestational age, growth retardation, congenital abnormalities and APGAR scores were documented. Cord pH was measured for all newborns with a delayed cry, Ambu bagging, intubation or pharmacological intervention. Any seizures, neurological problems, hypoglycaemia, hypothermia or hyperbilirubinaemia were assessed, diagnosed, evaluated and managed as per institutional guidelines. As per standard practice, newborns were screened for hearing deficits, vision and critical congenital heart disease according to institutional protocols. Preterm neonates transferred to the neonatal unit were monitored as per their customised follow-up and early intervention plans, implemented as recommended by the paediatrics and/or primary care provider. Neurodevelopment and biochemical assessment of preterm neonates were carried out as per their corrected gestational ages.

#### Infant follow-up

Infants were followed at 7–14 days; 4, 8, 12 and 16 weeks and at 6 and 9 months of corrected gestational age. Metabolic disorders (congenital hypothyroidism, congenital adrenal hyperplasia, cystic fibrosis, biotinidase deficiency, phenylketonuria and galactosaemia) were screened for and maternal support for breastfeeding was provided at 7–14 days by a paediatrician or lactation counsellor. Mother–infant dyads met a paediatrician for age-appropriate routine care, which involved clinical evaluation and preventive care including vaccination as per standard procedures in each site. Morbidities and recall of any illness by parents were recorded and treated as per institutional guidelines.

Anthropometric measurements and clinical signs of micronutrient deficiency (anaemia and rickets) were recorded. The infant’s weight was measured on a scale accurate to 5 g, and length was recorded with an infantometer precise to 0.1 cm, both taken in summer clothing using standard methods.

Any morbidities and deficiencies were recorded and treated as per institutional guidelines. Maternal and infant tolerance for the supplementation, including any gastrointestinal symptoms, was recorded at each visit, as was maternal compliance. Supplements were stopped for the mothers in both groups at 6 months postpartum. Infant interval morbidity was noted as any illness recalled by the parents in the period between two healthcare visits.

#### Infant assessment

The final assessment was conducted at 9 months (±14 days; extended to 12 months of age as described in online supplemental file). This included assessment of neurodevelopment as described below, infant blood sampling (2 mL in Serum Separating Tube), a complementary feeding assessment by a nutritionist using a 72-hour dietary intake diary and home environment assessment using the adapted Bradley HOME inventory^27^ by trained field staff.

#### Study outcomes

Infant neurodevelopment at 9–12 months of age (±14 days) using the Developmental Assessment Scales for Indian Infants was the primary outcome. This is an adapted, validated version of the Bayley’s Scale for Infant Development that has been in use in India and Nepal for more than four decades.^28^,^31^ Trained developmental therapists performed neurodevelopmental assessments at host institutes with caregivers present. Raw scores were converted into composite developmental quotient (DQ) motor and DQ mental scores. Therapists, co-trained at trial onset, achieved an ICC (Intra Class Coefficient) of 0.89 in joint assessments. During a pilot run (n=10), unsupervised therapist observations were video reviewed by others, yielding an ICC of 0.93. This process was repeated mid-trial with ten subjects and an ICC of 0.96 was recorded.

#### Secondary outcomes

- Change in B_12_ status (vitamin B_12_ level, Homocysteine and Holo-transcobalamin) of mother between first (<12 weeks’ gestation) and third trimester (>27 weeks’ gestation).
- B_12_ status (vitamin B_12_ level, Homocysteine and Holo-transcobalamin) of infants at 9 months (±2 weeks) post partum.

#### Exploratory outcomes

- Haemoglobin (Hb) of infants at 9 months (+14 days) post partum.
- Infant anthropometry, including weight, height/length and head circumference at 9 months after birth in all subjects.

##### Trial protocol modifications related to COVID-19

The trial protocol underwent midterm modifications, primarily in the context of the unanticipated COVID-19 pandemic. The maternal age eligibility was expanded from 35 to 40 years before the COVID-19 crisis to prevent exclusion of eligible participants (effective 25 November 2019). During the pandemic (from 13 August 2020), teleconsultations, external prescriptions, home-based services, increased capsule dispensing and extended infant follow-up to 12 months were permitted. The protocol modifications are more detailed in the online supplemental file.

### Sample size considerations

It was planned that 720 pregnant women, 360 per study site, would be randomly assigned to experimental (n=360; n=180 per study site) or control (n=360; n=180 per study site) groups. This was based on the fact that the number of infants needed to detect a difference in DQ at 9 months (primary outcome) was 2 points (presumed mean DQ in this population of infants at 9 months of 96.9, SD 7.07 on the basis of earlier work).^32^ The difference of 2 DQ points was chosen because it is the smallest effect size considered clinically significant.^32^ Hence, a null trial result will likely exclude a clinically relevant effect. The sample size was inflated by 35% to account for attrition and assumed 90% statistical power and a 5% two-sided significance level. The sample size estimation was done using the pwr package in R (R V.1.3-0).^33^

### Biochemical analysis

EDTA samples were processed by Coulter Counter at the study site. Serum samples were centrifuged at 3500 rpm (15 cm rotor radius) for 10 min, then stored at −80°C onsite. After study completion, samples were transported to the lab at −20°C and processed in one batch (SRL Reference Lab, Gurugram, India; NABL certified; EQAS validated). Maternal blood was analysed for Vitamin B12, Homocysteine, Holo Tc, 25(OH)D and folate during the first and third trimesters. Infant samples were tested for Hb, Vitamin B_12_, Homocysteine and Holo Tc. 25(OH)D, Ferritin, Folate and B_12_ were measured via Electro Chemiluminescence Immunoassay; Holo Tc by Chemiluminescent Microparticle Immunoassay; Homocysteine by Novel Enzyme Cycling. Coefficients of variation were: Vitamin B_12_ (3.5%), Homocysteine (2.4%), Holo Tc (4.9%), 25(OH)D (5.81%) and folate (7.03%).

Biochemical deficiencies in mothers were defined as anaemia: Hb <110 g/L; B_12_ Deficiency: Serum B_12_ <197 pg/mL; Hyperhomocystinaemia: Serum Homocysteine ≥12 µmol/L (with folate supplement), ≥15 µmol/L (without folate supplement); Holo TC Deficiency: Serum Holo TC <25.10 pmol/L; Low Ferritin: Ferritin <13 ng/mL; Folate Deficiency: Folate <4.4 ng/mL; Hypovitaminosis D: 25(OH)D<20 ng/mL. These cut-offs were laboratory-defined but align with internationally recognised ranges for deficiency and marginal status.^34^

#### Data analysis

Data were entered into a password-protected Microsoft Access database linked to a cloud drive in order to provide access to the designated researcher staff. Data files were checked manually and revalidated (double-checked) using the compareDF package in R.^35^ Data mining by classification, correlation, regression, outlier and subgroup analyses was used to identify any patterns of missing values.

All analyses followed an intention-to-treat approach. Continuous variables were summarised with descriptive statistics and plots; categorical variables by percentages. Group comparisons used Student’s t-test or analysis of variance for normal data, and Wilcoxon-Mann-Whitney for non-normal data. Normality was assessed with density plots and the Shapiro-Wilk test. Categorical proportions were compared using χ² tests. Analysis of covariance was applied to adjust for site differences. All statistical analyses were performed using R (R V.1.3-0)^33^ and were conducted without imputation of missing values as predefined in the protocol. All statistical analyses were performed using R (R V.1.3-0)^33^ and were conducted without imputation of missing values as predefined in the protocol. The study site, baby gender, breastfeeding status at 3 months, Weight for Age Z-score (score (WAZ) at birth, change in WAZ between birth to 3 months and the maternal body mass index at recruitment were the prespecified covariates used for calculating adjusted outcomes by linear regression. Subgroup analyses by Study Site, Maternal B12 deficiency status (<197 pg/mL), APGAR score of newborn (< 7 vs >=7 at 1 min), fetal growth retardation (<10 centile by WHO charts^26^ and high-risk maternal/infant categorisation as per prespecified in the study protocol.

## Results

**Figure 1:**
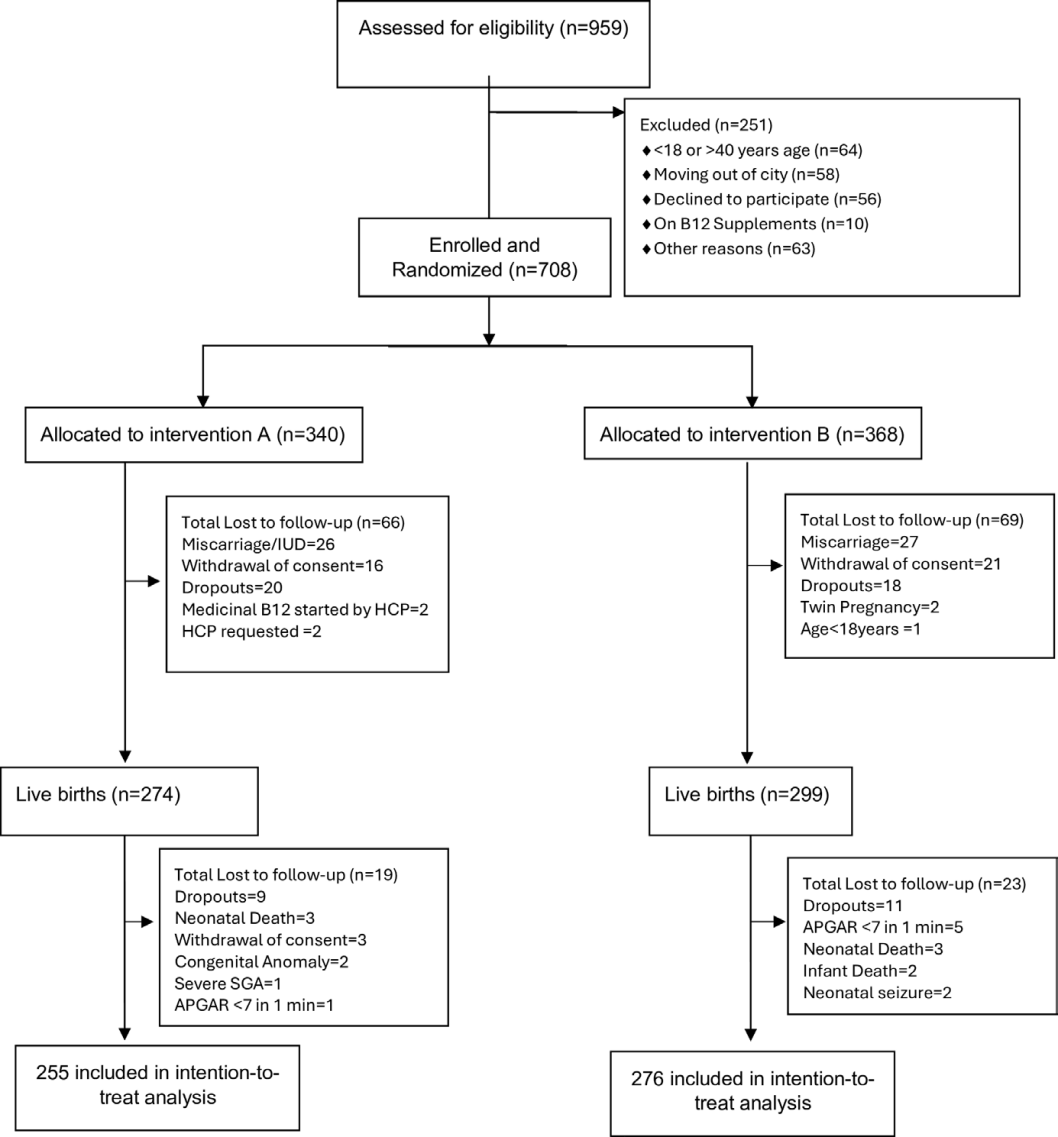
Trial flow diagram. HCP, healthcare provider; AGPAR, Appearance Pulse Grimace Activity & Respiration; SGA, Small for Gestational Age; IUD, Intra Uterine Death.

Figure 1 summarises participant flow. Of 959 women screened, 708 eligible participants were enrolled between 18 April 2019 and 19 April 2021. Overall, 531 mother–infant dyads (75%) completed follow-up to the 9–12-month assessment, and 177 participants (25%) were lost to follow-up; detailed reasons for exclusions and losses are provided in figure 1. Completion rates were similar across groups (group A: 255/340; group B: 276/368).

As presented in table 1, the two groups were comparable in terms of the sociodemographic variables, baseline. The two groups were also comparable for B_12_ status, 25(OH) D levels, serum folate and serum ferritin. It is noteworthy that 53.8% of the participants were deficient in B_12_, 75.9% were deficient in vitamin D, while 28.7% had low serum ferritin levels and 11.8% were anaemic in the first trimester. Mothers who completed the trial were comparable to those lost to follow-up for all sociodemographic characteristics (online supplemental table S1), although as expected, those lost to follow-up had a worse micronutrient profile compared with those who completed. The B_12_ intake of the mothers (assessed by FFQ) was comparable between the study groups (group A 2.86 µg/day vs group B 2.89 µg/day; p=0.654). Exclusive breastfeeding rates at 3 months ((n(%); group A 118 (95.2) vs group B 136 (97.1); p=0.400) and 6 months ((n(%); group A 91 (82.0) vs group B 110 (87.3); p=0.255) were comparable between the groups.

**Table 1:** Maternal baseline characteristics at enrolment

Characteristic	Overall (n=708)	Group A (250 µg/day) (n=340)	Group B (50 µg/day) (n=368)
Maternal sociodemographic/clinical			
Age, years (mean±SD)	28.08±4.64	27.92±4.72	28.24±4.58
Nuclear family, n (%)*	352 (49.8)	156 (45.9)	196 (53.4)
Pucca house, n (%)†	669 (94.6)	323 (95.0)	346 (94.3)
Education (graduation and above), n (%)‡	396 (56.0)	193 (56.8)	203 (55.3)
Profession (skilled and above), n (%)‡	441 (62.3)	215 (63.2)	226 (61.6)
Family income (INR/month), median (IQR)§	50 000 (18 750–120 000)	50 000 (18 750–110 000)	50 000 (18 750–122 500)
Primigravida, n (%)	418 (59.1)	208 (61.2)	210 (57.2)
Gestational age at recruitment, weeks (mean±SD)	9.00±1.94	9.14±1.94	8.88±1.94
Prepregnancy weight, kg (mean±SD)¶	57.38±10.44	57.38±10.57	57.39±10.35
Weight at recruitment, kg (mean±SD)¶	57.49±11.12	57.56±11.19	57.44±11.08
Height, cm (mean±SD)	156.87±6.05	157.10±5.86	156.67±6.22
Current smoking, n (%)	1 (0.1)	0 (0.0)	1 (0.3)
Current alcohol use, n (%)	6 (0.8)	3 (0.9)	3 (0.8)
Previous miscarriage, n (%)	179 (25.3)	82 (24.1)	97 (26.4)
Baseline biochemical profile (median (p25–75))**			
Hb, g/L	124.0 (115.0–130.0)	124.0 (116.0–131.0)	124.0 (115.0–130.0)
Serum vitamin B_12_, pg/mL	185.5 (136.0–262.0)	181.0 (136.0–251.0)	189.0 (135.5–271.0)
Homocysteine, µmol/L	10.0 (7.7–13.3)	10.0 (7.8–13.3)	10.0 (7.7–13.3)
Holotranscobalamin, pmol/L	28.6 (18.1–45.5)	27.6 (17.2–43.3)	29.3 (18.8–47.4)
Ferritin, ng/mL	23.3 (11.6 – 45.3)	23.0 (11.4 – 47.0)	23.5 (11.6–44.4)
Folate, ng/mL	20.0 (11.9–20.0)	20.0 (13.0–20.0)	20.0 (11.3–20.0)
Vitamin D, ng/mL	13.0 (8.9–19.7)	12.2 (8.6–19.0)	13.7 (9.3–20.1)
Baseline deficiency categories (selected), n (%)††			
Anaemia (Hb <110 g/L)‡‡	83 (11.8)	38 (11.2)	45 (12.3)
Vitamin B_12_ deficiency (serum B_12_ <197 pg/mL)§§	381 (53.8)	188 (55.5)	193 (52.9)
Hyperhomocystinaemia¶¶	221 (31.4)	108 (31.9)	113 (31.0)
Holo Tc deficiency***	298 (42.3)	145 (42.8)	153 (41.9)
Low ferritin†††	202 (28.7)	104 (30.7)	98 (26.8)
Folate deficiency‡‡‡	18 (2.6)	7 (2.1)	11 (3.0)
Hypovitaminosis D§§§	534 (75.9)	261 (77.0)	273 (74.8)

Continuous variables or number (percentage) for discrete variables; ANOVA test used for comparing groups for normally distributed continuous variables/Mann-Whitney for non-normally distributed variable and χ2 test for discrete variables. Mean differences and 95% CIs are provided only for normally distributed continuous variables.

* Nuclear family defined as parents living with their unmarried children in a house.

† Pucca house is brick and mortar.

‡ Collected and classified in accordance with the Kuppuswamy scale of socioeconomic status.^37^

§ Nepal data collected in NPR was converted to INR using a factor of 1.6:1.

¶ The prepregnancy weight is reported by the mother. It is not the same as the weight at recruitment which refers to the actual measurement taken at recruitment.

** OR median (p25–75).

†† Values represent mean ±SD.

‡‡ Anaemia: Hb <110g/L.

§§ B_12_ deficiency: Serum B_12_ <197 pg/mL.

¶¶ Hyperhomocystinaemia: Serum homocysteine ≥12 μmol/L (with folate supplement), ≥15 μmol/L (without folate supplement).

*** Holo Tc deficiency: Serum Holo Tc <25.10 pmol/L.

††† Low ferritin: Ferritin <13 ng/mL.

‡‡‡ Folate deficiency: Folate <4.4 ng/mL.

§§§ Hypovitaminosis D: 25(OH)D <20 ng/mL.

ANOVA, analysis of variance; Hb, haemoglobin; Holo Tc, holotranscobalamin.

Out of 531 infants, 359 (67.6%), 125 (23.5%), 34 (6.4%) and 13 (2.4%) were assessed at 9, 10, 11 and 12 months of age, respectively. Table 2 summarises the infant assessment at 9–12 months of age including the anthropometry, neurodevelopment and biochemical variables by ‘intention to treat’ analysis. At 9–12 months of age, infants of mothers in the 250 µg B_12_ group had a higher mean mental developmental quotient (DQ) than infants in the 50 µg group (103.7 vs 101.7, p=0.007). In contrast, motor DQ scores were similar between groups. Growth outcomes (weight, length and head circumference) did not significantly differ between the two groups. Infant vitamin B_12_ and holotranscobalamin levels at 9–12 months were comparable between groups, whereas infant homocysteine was lower in the 250 µg group.

**Table 2:** Infant outcomes at 9–12 months of age

Outcome	n	Group A (250 µg/day)	Group B (50 µg/day)	Effect estimate (A–B)	P value
Neurodevelopment outcomes*					
DASII mental DQ	531	103.7±7.73	101.7±8.8	1.94 (0.51–3.36)	**0.007**
DASII motor DQ	531	98.3±9.93	97.3±10.70	0.97 (−0.78–2.74)	0.277
DASII mental percentile	531	77.9±31.5	70.1±35.2	7.80 (2.10–13.50)	**0.007**
DASII motor percentile	531	49.8±30.7	46.8±30.9	3.05 (−2.20–8.30)	0.254
Infant vitamin B_12_ status (secondary outcomes; median, IQR)†					
Serum vitamin B_12_, pg/mL	482	371.0 (256.0–532.0)	352.0 (243.0–520.5)	—	0.469
Homocysteine, µmol/L	483	7.6 (6.6–9.8)	8.4 (6.4–10.6)	—	**0.009**
Holotranscobalamin, pmol/L	482	62.1 (34.1–128.0)	53.6 (33.6–127.9)	—	0.261
Infant exploratory outcomes*					
Haemoglobin, g/L	490	113.8±10.7	114.3±10.8	−0.50 (−2.40–1.40)	0.060
Weight, grams	509	8874±1028	8818±1103	55.22 (−126.90–237.40)	0.552
Weight-for-age Z score	531	0.18±0.93	0.16±1.01	0.02 (−0.14–0.19)	0.763
Length, cm	433	71.9±2.8	71.5±3.2	0.46 (−0.05–0.99)	0.080
Height-for-age Z score	527	0.27±1.19	0.10±1.28	0.16 (−0.04–0.37)	0.124
Head circumference, cm	435	44.7±1.63	44.5±1.6	0.16 (−0.10–0.44)	0.233
Infant micronutrient deficiencies (N (%))					
Anaemia‡	490	74 (31.4)	74 (29.0)	—	0.573
Vitamin B_12_ deficiency§	482	58 (25.1)	76 (30.2)	—	0.216
Hyperhomocystinaemia¶	483	54 (23.4)	76 (30.0)	—	0.099
Holo Tc deficiency**	482	29 (12.7)	39 (15.4)	—	0.396

For continuous variables; ANOVA test used for comparing groups for normally distributed continuous variables/Mann-Whitney for non-normally distributed variable. Mean differences and 95% CI are provided only for normally distributed continuous variables.

DASII mental and motor DQ scores are age-standardised by definition (developmental age/chronological age×100); corrected age was used for preterm infants, and chronological age for term infants; no additional age-correction was applied.

Bold entries refers to p<0.05.

* Values represent mean±SD.

† OR median (p25–p75).

‡ Anaemia: haemoglobin <110 g/L.

§ B_12_ deficiency: serum B_12_<259 pg/mL.

¶ Hyperhomocystinaemia: serum homocysteine ≥ µmol/L (with folate supplement), ≥10 µmol/L (without folate supplement).

** Holo Tc deficiency: serum Holo Tc <25.10 pmol/L.

ANOVA, analysis of variance; DASII, Developmental Assessment Scales for Indian Infants; DQ, developmental quotient; Holo Tc, holotranscobalamin.

Nutrient intake by complementary feeding (online supplemental table S2) and the home environment scores ((mean score±SD) (group A 35.4±3.6) vs group B 35.2±3.7); p=0.530) were also comparable between groups.

Online supplemental table S3a summarises the characteristics of the newborns who continued and those lost to follow-up. Online supplemental table S3b presents the babies born to the participants who continued in the study. The two groups were comparable for birth anthropometry, gestational age, APGAR score and neonatal morbidity.

The biochemical profile of the mothers in the third trimester analysed by ‘intention-to-treat’ analysis is summarised in table 3. The mean B_12_ and Holo Tc levels in group A were higher in the third trimester, while the homocysteine levels were lower. Serum ferritin, folate and 25(OH)D levels were comparable between the two groups at the third trimester. These differences corresponded to a decrease in B_12_ deficiency (low B_12_ levels) to 15.7% in group A vs 23.0% in group B by the third trimester, while low Holo Tc levels decreased to 6.0% and 3.8% in groups A and B, respectively. Meanwhile, the prevalence of anaemia increased to 28.9% in group A vs 28.3% in group B, iron deficiency (low ferritin) increased to 34.0% in group A vs 33.5% in group B, while vitamin D deficiency (low 25(OH)D levels) decreased to 53.2% in group A vs 49.0% in group B. Table 3 also compares the two groups for changes between third and first trimester. As presented, the B_12_ levels increased in both groups; by 105.0 (17.0 to 177.0) (median (p25 to p75)) pg/mL in group A and by 54.0 (−13.0 to 121.0) (median (p25 to p75)) pg/mL in group B (p<0.001). Similarly, the Holo Tc levels increased in group A by 47.4 (21.6 to 73.1) (median (p25 to p75)) pmol/L and by 32.9 (21.1 to 54.3) (median (p25 to p75)) in group B (p<0.001). Changes in homocysteine levels were not statistically different between the two groups (p=0.073). Also, changes in serum ferritin, folate and 25(OH)D levels were comparable between the two groups.

**Table 3:** Maternal micronutrient status: third trimester values and change from first trimester

Biomarker	n	Group A (250 µg/day)	Group B (50 µg/day)	P value
Third trimester biochemical parameters*				
Haemoglobin, g/L	455	115.5 (13.0)	114.7 (12.3)	0.474
Serum vitamin B_12_, pg/mL	474	293.0 (225.0–376.0)	264.0 (205.0–329.0)	**0.002**
Homocysteine, µmol/L	474	5.3 (4.4–6.6)	5.7 (4.7–6.9)	**0.036**
Holotranscobalamin, pmol/L	474	78.2 (52.2–116.5)	65.3 (49.2–96.6)	**0.004**
Ferritin, ng/mL	474	18.4 (11.1–31.8)	18.6 (10.6–32.7)	0.972
Folate, ng/mL	474	20.0 (16.9–20.0)	20.0 (16.2–20.0)	0.544
Vitamin D, ng/mL	474	19.0 (14.0–27.8)	20.2 (13.8–28.1)	0.615
Third trimester deficiency, n (%)				
Anaemia†	458	65 (28.9)	66 (28.3)	0.894
B_12_ deficiency‡	474	37 (15.7)	55 (23.0)	**0.045**
Hyperhomocystinaemia§	474	9 (3.8)	4 (1.7)	0.151
Holo Tc deficiency¶	474	14 (6.0)	9 (3.8)	0.267
Low ferritin**	474	80 (34.0)	80 (33.5)	0.896
Folate deficiency††	474	4 (1.7)	4 (1.7)	0.981
Hypovitaminosis D‡‡	474	125 (53.2)	117 (49.0)	0.356
Change from first trimester to third trimester*				
Serum vitamin B_12_, pg/mL	474	105.0 (17.0–177.0)	54.0 (−13.0–121.0)	**<0.001**
Homocysteine, µmol/L	474	−4.1 (−7.6–−2.1)	−3.7 (−6.3–−1.7)	0.073
Holotranscobalamin, pmol/L	474	47.4 (21.6–73.1)	32.9 (21.1–54.3)	**<0.001**
Ferritin (ng/mL)	474	−4.8 (−19.0–5.4)	−5.6 (−20.4–4.1)	0.670
Folate (ng/mL)	474	0.0 (0.0–4.3)	0.0 (0.0–4.0)	0.563
Vitamin D (ng/mL)	474	4.6 (−0.4–10.7)	4.4 (−1.0–11.7)	0.593

Bold entries refers to p<0.05.

* Values represent median (p25–p75); Mann−Whitney test used for comparing groups for non−normally distributed variables and χ2 test for discrete variables.

† Anaemia: haemoglobin <110 g/L.

‡ B_12_ deficiency: serum B_12_<197 pg/mL.

§ Hyperhomocystinaemia: serum homocysteine ≥12 µmol/L (with folate supplement), ≥15 µmol/L (without folate supplement).

¶ Holo Tc deficiency: serum Holo Tc <25.10 pmol/L.

** Low ferritin: Ferritin <13 ng/mL.

†† Folate deficiency: Folate <4.4 ng/mL.

‡‡ Hypovitaminosis D: 25(OH)D <20 ng/mL.

Holo Tc, holotranscobalamin.

Online supplemental table S4 presents that 87.2% of mothers were more than 60% compliant with B_12_ supplementation (>234 capsules taken out of 390 recommended) intake of B_12_ supplementation. There were no significant differences between the two groups. The incidences of intolerance and infant interval morbidity (table 4) were comparable between groups (maternal intolerance: 15 (4.4%) vs 7 (1.9%); p=0.054); Infant interval morbidity: 58 (17.1%) vs 65 (17.7%); p=0.832). No serious intervention-related adverse events occurred. There were 47 miscarriages and 8 neonatal deaths, with similar rates in both groups. Neonatal jaundice was the leading cause of newborn readmission. More details are provided in online supplemental table S5.

**Table 4:** Trial conduct, adherence and safety (including miscarriages and neonatal/infant deaths)

Measure	Group A (250 µg/day) n=340	Group B (50 µg/day) n=368
Adherence: ≥60% compliance (≥234 capsules)*, n (%)	226 (88.6)	237 (85.9)
Maternal intolerance†, n (%)	15 (4.4)	7 (1.9)
Infant interval morbidity†, n (%)	58 (17.1)	65 (17.7)
Miscarriages (pregnancy losses before delivery), n (%)	26 (7.6)	27 (7.3)
Neonatal/infant deaths, n	3 (0.9)	5 (1.4)

* Detailed compliance bands (60/70/80%) are shown in online supplemental table S4.

† Detailed maternal intolerance/infant interval morbidity categories are shown in online supplemental table S5.

Table 5 presents an analysis of the covariance of the motor and mental DQ using prespecified covariates. The adjusted difference in mental DQ was higher than the unadjusted difference (mean difference 3.8 vs 2.0). The motor DQ significantly differed between sites and correlated with WAZ at birth. There were no intergroup differences in motor DQ.

**Table 5:** Analysis of covariance: covariate adjusted outcomes

Predictor	Coefficient	95% CI (lower)	95% CI (upper)	P value
(A) Adjusted model for mental DQ				
Intercept	109.9	103.036	116.763	<0.001
Group (A–B)	3.806	1.791	5.82	<0.001
Study site (India–Nepal)	−0.004	−2.912	2.903	0.998
Infant sex (male–female)	−1.4	−3.39	0.591	0.167
Any breastfeeding at 3 months (yes–no)	−0.007	−3.069	3.055	0.996
WAZ at birth	0.426	−0.729	1.58	0.468
Change in WAZ (3 months−birth)	0.33	−0.829	1.5	0.578
Maternal BMI at recruitment (kg/m²)	−0.15	−0.414	0.114	0.265
(B) Adjusted model for motor DQ				
Intercept	104.981	96.981	112.981	<0.001
Group (A–B)	1.19	−1.158	3.539	0.319
Study site (India–Nepal)	−10.774	−14.163	−7.385	<0.001
Infant sex (male–female)	0.361	−1.959	2.681	0.76
Any breastfeeding at 3 months (yes–no)	0.159	−3.41	3.728	0.93
WAZ at birth	1.63	0.285	2.976	0.018
Change in WAZ (3 months−birth)	0.723	−0.64	2.087	0.297
Maternal BMI at recruitment (kg/m²)	0.031	−0.276	0.339	0.841

Models adjusted for prespecified covariates as per protocol; coefficients represent adjusted associations with DQ outcomes.

BMI, body mass index; DQ, developmental quotient; WAZ, weight for age Z-score.

To gain a deeper understanding of the findings, we performed the subgroup analyses by country, baseline maternal B_12_ deficiency status, APGAR score <7 at 1 min, SGA and high-risk status of the mother/infant as envisaged in the protocol. Table 6 presents the results for the subgroup analysis of B_12_-related parameters and infant neurodevelopment outcomes by country. Notably, significant differences in mental DQ scores were found only among Indian infants (104.4 vs 101.7; p=0.003 for India, compared with 103.0 vs 101.9; p=0.344 for Nepal). Online supplemental tables S6 and S7 provide comparisons of maternal baseline characteristics by country. Overall, Nepali participants tended to be younger, had lower socioeconomic status and recruitment weights, and experienced higher rates of deficiencies in B_12_, folate and vitamin D compared with Indian participants. Conversely, anaemia and iron deficiency were more common among Indian participants. Both groups showed improvements in B_12_ and iron status, with group A having a larger increase in B_12_—especially in Nepal; other nutrient changes were similar between groups and countries. Nepalese infants were generally smaller and shorter (see online supplemental table S8) but achieved higher motor DQ scores than Indian infants. The subgroup analyses for baseline maternal B_12_ deficiency status, APGAR score <7 at 1 min, SGA and high-risk status of the mother/infant are summarised as online supplemental table S9. The benefit in mental DQ appeared restricted to appropriate for gestational age (AGA), infants born to low-risk mothers/infants with an APGAR score >7 at 1 min while it was independent of baseline B_12_ status.

**Table 6:** Subgroup analysis by country (India vs Nepal): coprimary outcomes and key infant B_12_ biomarkers at 9–12 months

Outcome	Country	Overall*	Group A	Group B	P value†
DASII motor DQ‡	India	**93.9±9.2**	94.3±8.4	93.6±9.9	0.528
	Nepal	**102.3±9.7**	102.9±9.6	101.8±9.9	0.342
DASII mental DQ‡	India	103.0±7.5	104.4±6.1	101.7±8.4	**0.003**
	Nepal	102.5±9.3	103.0 (9.2)	101.9 (9.4)	0.344
Serum B_12_ (pg/mL)§¶	India	378.0 (251.1–564.0)	381.0 (265.5–556.0)	365.5 (241.0–591.0)	0.770
	Nepal	351.0 (246.0–492.5)	361.5 (247.3–515.3)	341.0 (243.0–476.0)	0.483
Homocysteine (µmol/L)**§	India	**7.3 (6.1–9.0)**	7.0 (5.9–8.6)	7.7 (6.2–9.3)	**0.045**
	Nepal	**8.9 (7.3–11.3)**	8.5 (7.1–10.4)	9.2 (7.4–11.7)	0.062
Holotranscobalamin (pmol/L)§††	India	**78.8 (41.2–128.0)**	95.6 (47.9–128.0)	68.3 (38.5–128.0)	0.241
	Nepal	**47.2 (28.3–81.3)**	45.5 (29.6–84.6)	47.3 (27.3–77.0)	0.724

* Bolded values in overall column represent significant differences between countries.

† P value represents differences between groups within country.

‡ Values represent mean (SD) or median (p25–p75)

§ For continuous variables; ANOVA test used for comparing groups for normally distributed continuous variables/Mann-Whitney for non-normally distributed variable and χ2 test for discrete variables.

¶ B_12_ deficiency: serum B_12_<259 pg/mL.

** Hyperhomocystinaemia: Serum Homocysteine ≥8 µmol/L (with folate supplement), 1≥0 µmol/L (without folate supplement).

†† Holo Tc deficiency: Serum Holo Tc <25.10 pmol/L.

ANOVA, analysis of variance; DASII, Developmental Assessment Scales for Indian Infants; DQ, developmental quotient; Holo Tc, holotranscobalamin.

## Discussion

Our study found that infants of vegetarian mothers supplemented with 250 µg/day of B_12_ had neurodevelopment scores about 2 DQ points higher than those receiving 50 µg. Both doses improved maternal B12 status, but 250 µg was more effective, with no safety concerns observed. The benefit was consistent after adjusting for covariates, and supplementation was particularly effective in Indian participants and low-risk pregnancies. The small difference in third-trimester total homocysteine, despite statistical significance, is unlikely to be clinically meaningful.

This large, multicentre, double-blind randomised controlled trial followed participants for approximately 17 months (covering pregnancy and postnatal periods), with a diverse sample in terms of socioeconomic status and ethnicity. The study’s strengths include its robust design, large sample size and focus on high-risk groups: vegetarian and pregnant women. It compared a higher pharmacological dose against a quasi-placebo, based on prior evidence that lower doses were ineffective. Limitations include assessment of only short-term infant neurodevelopment and restricted applicability to vegetarian pregnant women. The dropout rate was high (25%), largely due to natural events such as miscarriages and infant mortality, but adjusted to 13.9% after accounting for these factors; rates were comparable across groups and similar to community norms. Additional dropouts were related to COVID-19, blood sampling concerns and perceived lack of benefit. Loss to follow-up is unlikely to have biased results, as baseline characteristics and morbidity rates did not differ significantly between those retained and lost, and the sample size was preadjusted for expected attrition.

The results of the study are consistent with and add to some of the earlier work on the subject, even though the uniqueness in dose of supplementation, the quasi-placebo group and the selected population set preclude direct comparisons. The trial by Srinivasan *et al* showed that biochemical markers of B_12_ status did not improve with 50 µg/day while deteriorating with placebo in pregnancy.^18^ In comparison, our study documented improvement with both 250 µg/day and with 50 µg/day but greater improvement with 250 µg/day. Improvement in some domains of neurodevelopment with supplementation was noted in longer-term follow-up in the Srinivasan trial.^18^ While the intervention was not directly comparable, our study reports better neurodevelopment with higher dose supplementation. Another trial^20^ compared 2 µg/day of supplementation with/without multiple micronutrients with a placebo group after 5–8 years of supplementation through adolescence to pregnancy. Another trial^20^ compared 2 µg/day of supplementation with/without multiple micronutrients with a placebo group after 5–8 years of supplementation through adolescence to pregnancy. They reported a better biochemical maternal B_12_ status (at 28 weeks of gestation) and better neurodevelopment in babies (at 2 years) born to the supplemented groups. While no direct comparisons are possible, the results support the generic hypothesis that B_12_ supplementation leads to better maternal B_12_ status, which in turn improves infant neurodevelopment. In contrast, Chandyo *et al* reported a lack of improvement in neurodevelopment while reporting biochemical B_12_ improvement on maternal B_12_ supplementation with 50 µg.^21^ While the lack of a placebo group in our trial, lack of 250 µg/day group in the Nepal trial and different settings of intervention preclude direct comparisons, the findings by Chandyo *et al*^21^ are consistent with our a priori premise that 50 µg/day in pregnancy is ineffective. Our trial results also report biochemical improvement with 50 µg (same as Chandyo *et al*)^21^ and better neurodevelopmental score with 250 µg in comparison with 50 µg (no change in Chandyo *et al* with 50 µg). Our findings are consistent with the Nepal trial by Chandyo *et al*, which reported no improvement in infant neurodevelopmental outcomes despite biochemical improvements in maternal vitamin B_₁₂_ status. In our trial, when analyses were stratified by country, we similarly observed no statistically significant difference in infant neurodevelopment between the higher-dose and lower-dose B_₁₂_ groups among Nepalese participants. This pattern may suggest that, in the Nepal setting, factors such as coexisting micronutrient deficiencies and socioenvironmental influences (including the study site catering to lower income groups) could attenuate the neurodevelopmental impact of improved maternal B_₁₂_ status; however, these subgroup findings should be interpreted cautiously as the trial was not powered to detect site-specific effects. In contrast, the benefit observed in the Indian cohort may reflect differences in baseline nutritional status (less coexisting deficiencies) and contextual factors (study site catering to higher income population) that modify responsiveness to B_₁₂_ supplementation.

While a detailed review of the implications of all the above observations is beyond the current scope of study, these findings may be interpreted to reflect poor efficacy of B_12_ supplementation in the presence of a high prevalence of confounding multiple codeficiencies (like vitamin D and iron also affecting neurodevelopment) in lower-income subsets. Also, there could be a physiological basis for high pharmacological dose requirement when correction of severe deficiency is attempted in vegetarian populations. This could be related to genetically linked genetic polymorphisms in absorption or limitations in intrinsic factor-facilitated absorption in such settings.

Most research on B_12_ supplementation in pregnancy is observational, though some recent RCTs add to the evidence. While B_12_ supplements appear beneficial—especially for those with deficiencies—the evidence base is debated for public health recommendations.^36^ Our study stresses the need for guidelines on B_12_ for vegetarian pregnant women. Routine supplementation (500–1500 µg) is typically limited to those with private healthcare, as national programmes do not include it. With vegetarianism rising, especially in cities, clear expert guidance is urgently needed.

In summary, the study found that supplementing vegetarian pregnant women with 250 µg/day of B_12_ led to an approximate 2-point improvement in infant neurodevelopment compared with a quasi-placebo group receiving 50 µg/day. Longer-term studies and more detailed developmental assessments are needed to identify which subdomains benefit most and whether these effects persist. Further research should also examine physiological factors like intestinal absorption and use neuroimaging to clarify how B_12_ supplementation or deficiency affects different populations and regions.

## Supplementary material

10.1136/bmjpo-2025-004112 online supplemental file 1

## Data Availability

Data are available on reasonable request.

## References

[1] Vohr BR, Poggi Davis E, Wanke CA (2017). Neurodevelopment: The Impact of Nutrition and Inflammation During Preconception and Pregnancy in Low-Resource Settings. Pediatrics.

[2] Victora CG, Adair L, Fall C (2008). Maternal and child undernutrition: consequences for adult health and human capital. The Lancet.

[3] del Río Garcia C, Torres-Sánchez L, Chen J (2009). Maternal MTHFR 677C>T genotype and dietary intake of folate and vitamin B(12): their impact on child neurodevelopment. Nutr Neurosci.

[4] J Siddiqua T (2014). Vitamin B12 Deficiency in Pregnancy and Lactation: Is there a Need for Pre and Post-natal Supplementation?. *J Nutr Disorders Ther*.

[5] Dror DK, Allen LH (2008). Effect of vitamin B12 deficiency on neurodevelopment in infants: current knowledge and possible mechanisms. Nutr Rev.

[6] Bhate V, Deshpande S, Bhat D (2008). Vitamin B12 status of pregnant Indian women and cognitive function in their 9-year-old children. Food Nutr Bull.

[7] Torsvik I, Ueland PM, Markestad T (2013). Cobalamin supplementation improves motor development and regurgitations in infants: results from a randomized intervention study. Am J Clin Nutr.

[8] Black MM (2008). Effects of vitamin B12 and folate deficiency on brain development in children. Food Nutr Bull.

[9] Molloy AM, Kirke PN, Troendle JF (2009). Maternal vitamin B12 status and risk of neural tube defects in a population with high neural tube defect prevalence and no folic Acid fortification. Pediatrics.

[10] Refsum H (2001). Folate, vitamin B12 and homocysteine in relation to birth defects and pregnancy outcome. Br J Nutr.

[11] Sklar R (1986). Nutritional vitamin B12 deficiency in a breast-fed infant of a vegan-diet mother. Clin Pediatr (Phila).

[12] Kühne T, Bubl R, Baumgartner R (1991). Maternal vegan diet causing a serious infantile neurological disorder due to vitamin B12 deficiency. Eur J Pediatr.

[13] Allen LH (2009). How common is vitamin B-12 deficiency?. Am J Clin Nutr.

[14] Ulak M, Chandyo RK, Adhikari RK (2014). Cobalamin and folate status in 6 to 35 months old children presenting with acute diarrhea in Bhaktapur, Nepal. *PLoS One*.

[15] Taneja S, Bhandari N, Strand TA (2007). Cobalamin and folate status in infants and young children in a low-to-middle income community in India. Am J Clin Nutr.

[16] Ulak M, Kvestad I, Chandyo RK (2023). The effect of infant vitamin B_12_ supplementation on neurodevelopment: a follow-up of a randomised placebo-controlled trial in Nepal. Br J Nutr.

[17] Torsvik IK, Ueland PM, Markestad T (2015). Motor development related to duration of exclusive breastfeeding, B vitamin status and B12 supplementation in infants with a birth weight between 2000-3000 g, results from a randomized intervention trial. BMC Pediatr.

[18] Srinivasan K, Thomas T, Kapanee ARM (2017). Effects of maternal vitamin B12 supplementation on early infant neurocognitive outcomes: a randomized controlled clinical trial. Matern Child Nutr.

[19] Thomas S, Thomas T, Bosch RJ (2019). Effect of Maternal Vitamin B12 Supplementation on Cognitive Outcomes in South Indian Children: A Randomized Controlled Clinical Trial. Matern Child Health J.

[20] D’souza N, Behere RV, Patni B (2021). Pre-conceptional Maternal Vitamin B12 Supplementation Improves Offspring Neurodevelopment at 2 Years of Age: PRIYA Trial. Front Pediatr.

[21] Chandyo RK, Kvestad I, Ulak M (2023). The effect of vitamin B12 supplementation during pregnancy on infant growth and development in Nepal: a community-based, double-blind, randomised, placebo-controlled trial. Lancet.

[22] Rizzo G, Laganà AS, Rapisarda AMC (2016). Vitamin B12 among Vegetarians: Status, Assessment and Supplementation. Nutrients.

[23] Javid P, Christensen E (2016). Vegetarians are at high risk of vitamin B12 deficiency. Ugeskr Laeger.

[24] Duggan C, Srinivasan K, Thomas T (2014). Vitamin B-12 supplementation during pregnancy and early lactation increases maternal, breast milk, and infant measures of vitamin B-12 status. J Nutr.

[25] Nagpal J, Mathur MR, Rawat S (2020). Efficacy of maternal B_12_ supplementation in vegetarian women for improving infant neurodevelopment: protocol for the MATCOBIND multicentre, double-blind, randomised controlled trial. BMJ Open.

[26] Kiserud T, Piaggio G, Carroli G (2017). The World Health Organization Fetal Growth Charts: A Multinational Longitudinal Study of Ultrasound Biometric Measurements and Estimated Fetal Weight. PLoS Med.

[27] Bradley RH, Caldwell BM, Rock SL (1989). Home environment and cognitive development in the first 3 years of life: A collaborative study involving six sites and three ethnic groups in North America. Dev Psychol.

[28] Bindu Patni BP (2012). Developmental assessment scales for Indian infants (DASII).

[29] Metgud DC (2019). Concurrent Validity of the Gross Motor Component of Ages and Stages Questionnaire-3 with the Motor Scales of Developmental Assessment Scales for Indian Infants (DASII) in Risk Infants< 6 Months. Indian Journal of Physical Therapy and Research.

[30] Misra N, Pathak P (1996). Developmental Assessment Scales for Indian Infants (DASII): Manual.

[31] Madaan P, Saini L, Sondhi V (2021). Development Assessment Scale for Indian Infants: A Systematic Review and Perspective on Dwindling Cutoffs. Indian J Pediatr.

[32] Nagpal J, Rawat S (2024). A prospective cohort study to evaluate the utility of antenatal doppler velocimetry in prediction of fetal malnutrition, neonatal morbidity, mortality and neurodevelopmental outcome.

[33] Champely S, Ekstrom C, Dalgaard P (2020). Basic functions for power analysis. r package version 1.3-0. https://cran.r-project.org/web/packages/pwr/index.html.

[34] Refsum H, Yajnik CS, Gadkari M (2001). Hyperhomocysteinemia and elevated methylmalonic acid indicate a high prevalence of cobalamin deficiency in Asian Indians. Am J Clin Nutr.

[35] Joseph A (2021). compareDF: Do a Git Style Diff of the Rows Between Two Dataframes with Similar Structure. R Package Version.

[36] Kurpad AV, Singh Sachdev H (2023). Efficacy of maternal vitamin B12 supplementation for improving infant outcomes in settings with high deficiency. The Lancet.

[37] Singh T, Sharma S, Nagesh S (2017). Socio-economic status scales updated for 2017. *Int J Res Med Sci*.

