# Supplementary material for: Maternal supplementation of vitamin B12 in predominantly vegetarian pregnant women improves their vitamin B12 status and the neurodevelopment of their infants: the MATCOBIND multicentric double-blind randomised control trial
Source: BMJ Paediatr Open. 2026 Mar 18;10(1):e004112. doi: 10.1136/bmjpo-2025-004112 (PMC13034390; doi:10.1136/bmjpo-2025-004112)
Supplement: online supplemental file 1 [file bmjpo-10-1-s001.docx]

**Supplementary File**

Annexure 1: COVID Modifications

The inclusion age of mother was increased from 35 to 40 years for enrolment. This modification was done before the COVID 19 crisis because a significant proportion of the mothers were in the said age group and were being excluded from the trial (with effect from 25/11/2019).

During the COVID-19 pandemic (with effect from 13/08/2020)

- - Teleconsultation was considered as a valid assessment visit for the trial during the pandemic
  - Prescription data by practitioners outside the study institutions was considered valid
  - The number of capsules dispensed was changed to 60 capsules, from 30 capsules and home delivery was allowed 3rd trimester blood sampling was done at home for patients not wanting to visit the trial site
  - Infant follow up age bracket was extended up to 12 months

**Supplementary Table S1. Baseline characteristics of participants who completed follow-up vs were lost to follow-up**

| **Characteristics** | **N** | **Overall**  (n=708) | **Completed Follow up**  (n=531) | **Lost to Follow Up**  (n=177) | **95% CI**  **Lower** | **95% CI**  **Upper** |
| --- | --- | --- | --- | --- | --- | --- |
| **Maternal Socio-demographic Characteristics** | | | | | | |
| Age, years (mean ± SD) | 708 | 28.1 ± 4.6 | 28.1 ± 4.6 | 28.0 ± 4.8 | -0.69 | 0.89 |
| Nuclear family, n (%)^#^ | 707 | 352 (49.8) | 263 (49.5) | 89 (50.6) | - | - |
| Pucca house, n (%)^$^ | 707 | 669 (94.6) | 504 (94.9) | 165 (93.8) | - | - |
| Education (graduation and above), n (%)^Y^ | 707 | 396 (56.0) | 299 (56.3) | 97 (55.1) | - | - |
| Profession (skilled and above), n (%)^Y^ | 707 | 441 (62.4) | 332 (62.5) | 109 (61.9) | - | - |
| Family income (INR/month), ^μ I^ | 692 | 50000.0 (18750.0 to 120000.0) | 50000.0 (19000.0 to 120000.0) | 37500.0 (18750.0 to 100000.0) | - | - |
| Primigravida, n (%) | 707 | 418 (59.1) | 318 (59.9) | 100 (56.8) | - | - |
| Gestational age at recruitment, weeks (mean ± SD) | 707 | 9.0 ± 1.9 | 9.1 ± 1.9 | 8.8 ± 1.9 | -0.03 | 0.63 |
| Pre-pregnancy weight, kg (mean ± SD)^ | 707 | 57.4 ± 10.4 | 57.4 ± 10.6 | 57.3 ± 9.9 | -1.73 | 1.83 |
| Weight at recruitment, kg (mean ± SD)^ | 706 | 57.5 ± 11.1 | 57.5 ± 11.3 | 57.6 ± 10.5 | -2.03 | 1.77 |
| Height, cm (mean± SD) | 707 | 156.9 ± 6.1 | 156.8 ± 6.1 | 157.1 ± 6.0 | -1.37 | 0.69 |
| Current smoking, n (%) | 707 | 1 (0.1) | 1 (0.2) | 0 (0.0) | - | - |
| Current alcohol use, n (%) | 707 | 6 (0.8) | 5 (0.9) | 1 (0.6) | - | - |
| Previous miscarriage, n (%) | 707 | 179 (25.3) | 132 (24.9) | 47 (26.7) | - | - |
| **Maternal Biochemical Parameters at First trimester^μ^** | | | | | | |
| Haemoglobin (g/L) | 692 | 124.0 (115.0 to 130.0) | 123.0 (116.0 to 129.0) | 125.0 (115.0 to 133.0) | - | - |
| Serum B12 (pg/mL) | 692 | 184.0 (135.3 to 261.5) | 187.0 (140.0 to 266.0) | 171.0 (126.5 to 229.5) | - | - |
| Homocysteine (μmol/L) | 692 | 10.0 (7.8 to 13.3) | 9.6 (7.6 to 12.9) | 11.1 (8.6 to 14.8) | - | - |
| Holo Tc(pmol/L) | 692 | 28.4 (17.8 to 44.8) | 29.4 (18.6 to 47.2) | 24.9 (16.6 to 37.0) | - | - |
| Ferritin (ng/mL) | 692 | 23.4 (11.6 to 45.4) | 24.5 (12.7 to 46.9) | 18.0 (10.2 to 42.0) | - | - |
| Folate (ng/mL) | 692 | 20.0 (11.9 to 20.0) | 20.0 (12.0 to 20.0) | 19.9 (11.8 to 20.0) | - | - |
| Vitamin D (ng/mL) | 692 | 12.9 (8.9 to 19.4) | 13.1 (9.1 to 19.9) | 12.1 (8.3 to 18.1) | - | - |
| **Micronutrient Deficiencies, n (%)** | | | | | | |
| Anaemia^a^ | 704 | 83 (11.8) | 60 (11.3) | 23 (13.3) | - | - |
| B12 Deficiency^b^ | 704 | 381 (53.8) | 278 (52.4) | 103 (58.2) | - | - |
| Hyperhomocystinemia^c^ | 704 | 221 (31.4) | 155 (29.2) | 66 (38.2) | - | - |
| Holo Tc Deficiency^d^ | 704 | 298 (42.3) | 211 (39.7) | 87 (50.3) | - | - |
| Low ferritin^e^ | 704 | 202 (28.7) | 137 (25.8) | 65 (37.6) | - | - |
| Folate deficiency^f^ | 704 | 18 (2.6) | 11 (2.1) | 7 (4.0) | - | - |
| Hypovitaminosis D^g^ | 704 | 534 (75.9) | 397 (74.8) | 137 (79.2) | - | - |

Values represent Mean (SD)^*^ or median (p25-75)^μ^ Continuous variables or Number [Percentage] for discrete variables; ANOVA test used for comparing groups for normally distributed continuous variables/Mann Whitney for non-normally distributed variable and Chi-Square test for discrete variables. Mean differences and 95% CI are provided only for normally distributed continuous variables.

^#^Nuclear family defined as parents living with their unmarried children in a house.

^$^Pucca house is brick and mortar.

^Y^Collected and classified in accordance with the Kuppuswamy scale of Socio-economic status

^I^Nepal data collected in NPR was converted to INR using a factor of 1.6:1

^The pre-pregnancy weight is reported by the mother. It is not the same as the weight at recruitment which refers to the actual measurement taken at recruitment.

^a^Anaemia: Hemoglobin <110g/L

^b^Maternal B12 Deficiency: Serum B12 <197pg/ml

^c^Maternal Hyperhomocystinemia: Serum Homocysteine =>12μmol/L (with folate supplement), ≥15 μmol/L (without folate supplement

^d^Holo Tc Deficiency: Serum Holo TC <25.10pmol/L

^e^Low Ferritin: Ferritin <13ng/mL

^f^Folate Deficiency: Folate <4.4ng/mL

^g^Hypovitaminosis D: 25(OH)D <20ng/mL

**Supplementary Table S2: Infant nutrient intake by complementary feeding (72-hour dietary diary)**

| **S.No.** | **Nutrients** | ***N*** | **Overall (*N=*524)** ^μ^ | **Group A (*n=*252)** ^μ^ | **Group B (*n=*272)** ^μ^ | ***p-*value** |
| --- | --- | --- | --- | --- | --- | --- |
| 1. | Energy (KCal) | 524 | 562.27 (349.77 to 1487.65) | 550.75 (345.71 to 1468.87) | 568.87 (349.99 to 1500.00) | 0.831 |
| 2. | Protein (g) | 524 | 29.66 (10.82 to 55.75) | 21.62 (10.52 to 55.76) | 20.10 (11.15 to 55.74) | 0.822 |
| 3. | Fat (g) | 524 | 10.93 (6.21 to 17.78) | 10.96 (5.85 to 17.52) | 10.90 (6.70 to 18.40) | 0.721 |
| 4. | Carb (g) | 524 | 85.81 (55.16 to 210.38) | 85.23 (54.76 to 210.50) | 86.05 (55.27 to 210.38) | 0.946 |
| 5. | Iron (mg) | 524 | 4.30 (2.39 to 38.23) | 4.53 (2.34 to 38.42) | 4.25 (2.40 to 37.74) | 0.587 |
| 6. | Calcium (mg) | 524 | 222.20 (90.24 to 662.32) | 221.90 (89.92 to 674.39) | 223.08 (90.24 to 659.87) | 0.523 |
| 7. | Vit B12 (μg) | 383 | 0.31 (0.10 to 0.52) | 0.29 (0.07 to 0.44) | 0.33 (0.12 to 0.54) | 0.285 |
| 8. | Folic Acid (μg) | 524 | 50.13 (31.15 to 114.57) | 45.50 (30.17 to 103.58) | 53.43 (33.00 to 132.75) | 0.337 |
| 9. | Vit D (IU) | 524 | 1.66 (0.55 to 10.50) | 2.25 (0.45 to 7.50) | 1.50 (0.55 to 12.00) | 0.883 |

Values represent median (p25-75)^μ^ for continuous variables; Mann Whitney test for used for comparing non-normally distributed variable

**Supplementary Table S3**

| **Table S3a: Neonatal Characteristics, included vs Loss to Follow Infants (*n=*575 of 708; mean (SD) or**  ***n (%)*)** | | | | | | | | |
| --- | --- | --- | --- | --- | --- | --- | --- | --- |
| **Characteristics** | ***N*** | **Overall (*n=*575)** | **Continued in follow up(*n=*531)** | **Loss to follow (*n=*44)** | **Mean Diff** | **Difference 95% CI** | | ***p-value*** |
| Birth Weight (grams) mean ± SD | 551 | 3017.7 ±436.8 | 3026.5 ±427.7) | 2911.4 ±530.1 | 115.09 | -22.44 | 252.64 | 0.101 |
| Birth Length (cm) mean ± SD | 463 | 48.9 ±2.3 | 48.9 ±2.3) | 48.6 ±2.3 | 0.27 | -0.58 | 1.13 | 0.533 |
| APGAR at 1 min mean ± SD | 476 | 7.3 ±1.5 | 7.4 ±1.4) | 6.6 ±2.1 | 0.82 | 0.32 | 1.33 | **0.001** |
| APGAR at 5 min mean ± SD | 477 | 8.4 ±0.9 | 8.4 ±0.8) | 7.6 ±1.9 | 0.15 | 0.56 | 1.17 | **<0.001** |
| Gender (n, male %) | 575 | 286 (49.7) | 260 (49.0) | 26 (59.1) | - | - | - | 0.197 |
| SGA (n, yes %) | 575 | 16 (2.8) | 14 (2.6) | 2 (4.50 | - | - | - | 0.076 |
| Neonatal morbidity (n, yes%) | 575 | 125 (21.7) | 107 (20.2) | 18 (40.9) | - | - | - | **0.001** |
| **Table S3b: Neonatal Characteristics of subjects continuing follow up (*n=*531 of 708; mean (SD) or *n (%)*)** | | | | | | | | |
| **Characteristics** | ***N*** | **Group A (250mg; Intervention; *n=*255)** | **Group B**  **(50mg; Quasi-control; *n=*276)** | **Mean  Difference** | | **95% CI of Difference** | | ***p-value*** |
| Birth Weight, grams  mean ± SD | 509 | 3043.1± 441.5 | 3010.7± 414.3 | 32.33 | | -42.2 | 106.8 | 0.394 |
| Birth Length, cm  mean ± SD | 433 | 48.9± 2.4 | 48.8± 2.2 | 0.056 | | -0.38 | 0.49 | 0.799 |
| APGAR at 1 min  mean ± SD | 442 | 7.38± 1.3 | 7.3± 1.3 | -0.006 | | -0.262 | 0.25 | 0.963 |
| APGAR at 5 min  mean ± SD | 441 | 8.42± 0.7 | 8.4± 0.7 | -0.013 | | -0.155 | 0.128 | 0.853 |
| Gender (n, male %) | 531 | 135(52.9) | 124(44.9) | - | | - | - | 0.123 |
| SGA (n, yes %) | 531 | 6(2.4) | 8(2.9) | - | | - | - | 0.208 |
| Neonatal morbidity (n, yes %) | 531 | 52(20.4) | 55(19.9) | - | | - | - | 0.894 |

APGAR: Appearance, Pulse, Grimace, Activity, Respiration

SGA: Small for Gestation Age

*Values represent Mean (SD) for continuous variables or Number (Percentage) for discrete variables; ANOVA test used for comparing groups for continuous variables and Chi-Square test for discrete variables

**Supplementary Table S4. Vitamin B12 supplementation compliance**

| **S.No.** | **Compliance (%)** | **Overall (n=531) N (%)** | **Group A (n=255) N (%)** | **Group B  (n=276) N (%)** | **p-value** |
| --- | --- | --- | --- | --- | --- |
| 1. | ≥234 capsules (60%) | 463 (87.20) | 226 (88.60) | 237 (85.90) | 0.637 |
| 2. | ≥ 273 capsules (70%) | 389 (73.30) | 190 (74.50) | 199 (72.10) | 0.822 |
| 3. | ≥ 312 capsules (80%) | 272 (51.20) | 128 (50.20) | 144 (52.20) | 0.901 |

*390 capsules to be consumed in antenatal period and 6month postpartum

Number (Percentage) for discrete variables; Chi-Square test was used for comparing discrete variables

**Supplementary Table S5. Maternal intolerance and infant interval morbidity**

| **S.No.** | **Supplements intolerance** | **Overall  (n=708) N (%)** | **Group A  (n=340) N (%)** | **Group B (n=368) N (%)** | **p-value** |
| --- | --- | --- | --- | --- | --- |
| **I** | **Mother** |  |  |  |  |
| 1. | Any morbidity | 22 (3.1) | 15 (4.4) | 7 (1.9) | 0.054 |
| a. | Itching | 15 (2.1) | 9 (2.6) | 6 (1.6) | 0.348 |
| b. | Rash | 4 (0.6) | 3 (0.9) | 1 (0.3) | 0.279 |
| c. | Quit/Exited trial | 3 (0.4) | 3 (0.9) | 0 (0.0) | 0.110 |
| **II** | **Infant** |  |  |  |  |
| 1. | Any morbidity | 123 (17.4) | 58 (17.1) | 65 (17.7) | 0.832 |
| a. | Acute Respiratory Illness | 51 (7.2) | 26 (7.6) | 25 (6.8) | 0.661 |
| b. | Diarrhoea | 24 (3.4) | 13 (3.8) | 11 (3.0) | 0.540 |
| c. | Colic issues | 7 (1.0) | 2 (0.6) | 5 (1.4) | 0.301 |
| d. | COVID positive | 10 (1.4) | 3 (0.9) | 7 (1.9) | 0.251 |
| e. | Urticaria | 3 (0.4) | 1 (0.3) | 2 (0.5) | 0.610 |
| f. | Acute Illness | 7 (1.0) | 3 (0.9) | 4 (1.1) | 0.783 |
| g. | Reflux/Vomiting | 3 (0.4) | 0 (0.0) | 3 (0.8) | 0.095 |
| g. | Constipation | 2 (0.3) | 0 (0.0) | 2 (0.5) | 0.173 |
| i. | Neonatal Jaundice | 16 (2.3) | 10 (2.9) | 6 (1.6) | 0.241 |

Number (Percentage) for discrete variables; Chi-Square test was used for comparing discrete variables

**Supplementary Table S6: Sub-Group Analysis: Country-Wise and Group by Country comparison of Maternal Baseline Characteristics**

| **Characteristics** | **Overall India (*n=*370)** | **Overall Nepal (*n=*338)** | ***p-value*** | **INDIA (*n=*370)** | | ***p-value*** | **NEPAL**  **(*n=*338)** | | ***p-value*** |
| --- | --- | --- | --- | --- | --- | --- | --- | --- | --- |
|  |  |  |  | **Group A** | **Group B** |  | **Group A** | **Group B** |  |
| **Mother Characteristics** | | | | | | | | | |
| Age, years (mean (SD)) | 30.5(3.4) | 25.4(4.3) | **<0.001** | 30.5(3.5) | 30.5(3.3) | 0.994 | 24.9(4.1) | 25.8(4.4) | 0.068 |
| Nuclear Family type (*n (%)*) | 152(41.1) | 200(59.2) | **<0.001** | 69(38.1) | 83(43.9) | 0.500 | 87(54.7) | 113(63.1) | **0.044** |
| Pucca House (*n (%)*) | 369(99.7) | 300(88.8) | **<0.001** | 181(100) | 188(99.47) | - | 142(89.3) | 158(88.3) | 0.205 |
| Education (Graduation and above) (*n (%)*)^Y^ | 359(97.0) | 59(17.5) | **<0.001** | 179(98.9) | 180(95.2) | 0.142 | 27(15.1) | 29(16.2) | 0.601 |
| Profession (n, skilled above%) ^Y^ | 205(55.4) | 27(8.0) | **<0.001** | 96(53.04) | 109(57.7) | 0.490 | 12(7.6) | 15(8.4) | 0.006 |
| Family Income (in INR/month) (mean (SD)) ^l^ | 142966.5(104601.0) | 27041.4(26527.9) | **<0.001** | 133954.5(95809.9) | 151681.3(112026.4) | 0.109 | 28710.69(31276.9) | 25558.7(21431.5) | 0.276 |
| Primigravida (*n (%)*) | 199(53.9) | 219(64.8) | **0.003** | 93(51.4) | 106(56.4) | 0.335 | 115(72.3) | 104(58.1) | **0.006** |
| Gestational age, weeks at recruitment (mean (SD)) | 8.7(2.2) | 9.4(1.6) | **<0.001** | 8.9(2.2) | 8.4(2.0) | **0.027** | 9.3(1.4) | 9.3(1.6) | 0.884 |
| Pre-pregnancy weight, kg (mean (SD)) | 62.0(10.1) | 52.3(8.3) | **<0.001** | 62.1(10.2) | 61.9(9.9) | 0.930 | 52.0(8.1) | 52.5(8.3) | 0.545 |
| Recruitment weight (kg) (mean (SD)) | 62.9(10.3) | 51.7(8.8) | **<0.001** | 62.9(10.3) | 62.8(10.3) | 0.952 | 51.4(8.7) | 51.8(8.8) | 0.734 |
| Height, cm (mean (SD)) | 159.3(5.5) | 154.2(5.4) | **<0.001** | 159.2(5.5) | 159.3(5.5) | 0.833 | 154.6(5.2) | 153.8(5.5) | 0.168 |
| Currently Smoking (*n (%)*) | 0(0.0) | 1(0.3) | **0.04** | 0(0) | 0(0) | 0.314 | 0(0) | 1(0.6) | **0.006** |
| Current Alcohol consuming status (*n (%)*) | 0(0.0) | 6(1.8) | **<0.001** | 0(0) | 0(0) | 0.936 | 3(1.9) | 3(1.7) | 0.916 |
| Previous miscarriage (*n (%)*) | 116(31.4) | 63(18.6) | **<0.001** | 58(32.0) | 58(30.9) | 0.805 | 24(15.1) | 39(21.8) | 0.115 |
| ***Mother*, Median (p25 to p75)** | | | | | | | | | |
| Haemoglobin (g/L) | 121.5 (113.8 to 127.0) | 127.0 (118.0 to 133.3) | **<0.001** | 122.0 (114.0 to 129.0) | 120.0 (113.0 to 126.0) | 0.143 | 127.0 (118.0 to 134.0) | 127.0 (117.0 to 133.0) | 0.847 |
| Serum B12 (pg/mL)^m^ | 204.0 (148.8 to 291.5) | 170.0 (123.8 to 226.3) | **<0.001** | 201.0 (150.0 to 265.0) | 209.0 (144.0 to 304.0) | 0.545 | 164.0 (121.0 to 227.0) | 180.0 (126.0 to 226.0) | 0.376 |
| Homocysteine (μmol/L)^m^ | 9.2 (7.2 to 11.7) | 10.9 (8.5 to 15.4) | **<0.001** | 9.0 (7.2 to 11.8) | 9.5 (7.2 to 11.5) | 0.795 | 11.3 (8.8 to 15.2) | 10.8 (8.3 to 15.6) | 0.226 |
| Holo Tc(pmol/L)^m^ | 33.3 (21.1 to 52.8) | 24.8 (14.8 to 36.6) | **<0.001** | 31.8 (21.1 to 49.7) | 33.8 (21.0 to 57.7) | 0.479 | 23.8 (14.6 to 34.9) | 25.6 (15.4 to 37.5) | 0.322 |
| Ferritin (ng/mL)^m^ | 15.4 (8.9 to 27.3) | 37.1 (18.6 to 62.9) | **<0.001** | 15.6 (9.4 to 27.2) | 15.2 (8.5 to 27.9) | 0.608 | 37.9 (18.0 to 65.4) | 37.1 (18.8 to 59.0) | 0.938 |
| Folate (ng/mL)^m^ | 20.0 (14.5 to 20.0) | 15.5 (10.7 to 20.0) | **<0.001** | 20.0 (16.7 to 20.0) | 20.0 (14.2 to 20.0) | 0.266 | 16.0 (11.5 to 20.0) | 15.5 (10.6 to 20.0) | 0.407 |
| Vitamin D (ng/mL)^m^ | 15.7 (9.5 to 25.7) | 11.5 (8.3 to 15.8) | **<0.001** | 14.9 (9.2 to 25.2) | 16.4 (10.2 to 27.1) | 0.356 | 10.9 (8.0 to 15.0) | 12.2 (8.6 to 15.9) | 0.086 |
| **Micronutrient Deficiencies, *n (%)*** | | | | | | | | | |
| Anaemia^a^ | 56 (15.3) | 27 (8.0) | **0.003** | 25 (13.9) | 31 (16.7) | 0.461 | 13 (8.2) | 14 (7.8) | 0.904 |
| B12 Deficiency^b^ | 171 (46.7) | 210 (62.1) | **<0.001** | 86 (47.5) | 85 (45.0) | 0.624 | 102 (64.2) | 108 (60.3) | 0.470 |
| Hyperhomocystinemia^c^ | 77 (21.0) | 144 (42.6) | **<0.001** | 38 (21.1) | 39 (21.0) | 0.973 | 70 (44.0) | 74 (41.3) | 0.618 |
| Holo Tc Deficiency^d^ | 125 (34.2) | 173 (51.2) | **<0.001** | 60 (33.3) | 65 (34.9) | 0.745 | 85 (53.5) | 88 (49.2) | 0.430 |
| Low ferritin^e^ | 154 (42.1) | 48 (14.2) | **<0.001** | 77 (42.8) | 77 (41.4) | 0.789 | 27 (17.0) | 21 (11.7) | 0.168 |
| Folate deficiency^f^ | 12 (3.3) | 6 (1.8) | 0.207 | 4 (2.2) | 8 (4.3) | 0.264 | 3 (1.9) | 3 (1.7) | 0.884 |
| Hypovitaminosis D^g^ | 225 (61.5) | 309 (91.4) | **<0.001** | 113 (62.8) | 112 (60.2) | 0.615 | 148 (93.1) | 161 (89.9) | 0.304 |

*Values represent Mean (SD) or median (p25 to p75) ^μ^  for continuous variables or Number [Percentage] for discrete variables; ANOVA test used for comparing groups for normally distributed continuous variables/Mann Whitney for non-normally distributed variable and Ch-Square test for discrete variables. Mean differences and 95% CI are provided only for normally distributed continuous variables.

Pucca house is brick and mortar.

Nuclear family defined as parents living with their unmarried children in a house.

^Y^Collected and classified in accordance with the Kuppuswamy scale of Socio-economic status^30^

^l^Nepal data collected in NPR was converted to INR using a factor of 1.6:1

^a^Anaemia: Hemoglobin <110g/L

^b^B12 Deficiency: Serum B12 <197pg/ml

^c^Hyperhomocystinemia: Serum Homocysteine ≥ 12μmol/L (with folate supplement), ≥ 15 μmol/L (without folate supplement)

^d^Holo Tc Deficiency: Serum Holo TC <25.10pmol/L

^e^Low Ferritin: Ferritin <13ng/mL

^f^Folate Deficiency: Folate <4.4ng/mL;

^g^Hypovitaminosis D: 25(OH)D <20ng/mL

**Supplementary Table S7: Sub-Group Analysis: Country-Wise and Group by Country comparison of Changes in Maternal Biochemical Parameters At 3^rd^ trimester**

| **Characteristics** | **India** | **Nepal** | ***p-value*** | **INDIA  (*n=*283)** | | ***p-value*** | **NEPAL  (*n=*248)** | | ***p-value*** |
| --- | --- | --- | --- | --- | --- | --- | --- | --- | --- |
|  |  |  |  | **Group A (*n=*135)** | **Group B (*n=*148)** |  | **Group A (*n=*120)** | **Group B  (*n=*128)** |  |
| **Biochemical Parameters at Third trimester, Mean** ± **SD** | | | | | | | | | |
| Haemoglobin (g/L) | 111.3 ± 10.7 | 119.5 ± 13.4 | **<0.001** | 111.0 ± 10.2 | 111.6 ± 11.1 | 0.648 | 120.6 ±13.9 | 118.4 ± 12.7 | 0.245 |
| Ferritin (ng/mL) ^μ^ | 17.3 ± 13.8 | 33.7 ± 23.2 | **<0.001** | 17.3±13.4 | 17.4±14.3 | 0.972 | 32.3±21.6 | 35.2±24.8 | 0.355 |
| Serum B12 (pg/mL) ^μ^ | 295.0±121.5 | 305.6±155.4 | 0.406 | 312.5±138.9 | 278.5±100.2 | **0.024** | 332.4±184.5 | 277.6±112.1 | **0.010** |
| Homocysteine (μmol/L) ^μ^ | 5.9±1.9 | 6.2±2.8 | 0.276 | 5.8±2.1 | 6.0±1.8 | 0.379 | 6.2±3.3 | 6.1±2.3 | 0.872 |
| Holo Tc (pmol/L) ^μ^ | 83.3±42.6 | 76.9±42.6 | 0.106 | 88.7±46.3 | 78.1±38.2 | **0.044** | 83.9±46.8 | 69.6±36.6 | **0.014** |
| Folate (ng/mL) ^μ^ | 18.2±3.9 | 16.7±4.8 | **<0.001** | 18.4±3.9 | 18.0±3.9 | 0.428 | 16.8±4.6 | 16.7±5.0 | 0.885 |
| Vitamin D (ng/mL) ^μ^ | 26.3±13.2 | 17.2±7.1 | **<0.001** | 26.4±12.6 | 26.3±13.8 | 0.919 | 16.5±6.6 | 17.9±7.5 | 0.144 |
| **Deficiencies, *n (%)*** | | | | | | | | | |
| Anaemia^a^ | 96 (39.2) | 35 (16.4) | **<0.001** | 49 (41.5) | 47 (37.0) | 0.469 | 16 (15.0) | 19 (17.9) | 0.558 |
| B12 Deficiency^b^ | 43 (16.4) | 49 (23.1) | 0.067 | 18 (14.2) | 25 (18.5) | 0.343 | 19 (17.6) | 30 (28.8) | **0.052** |
| Hyperhomocystinemia^c^ | 2 (0.8) | 11 (5.2) | **0.003** | 2 (1.6) | 0 (0.0) | 0.143 | 7 (6.5) | 4 (3.8) | 0.387 |
| Holo Tc Deficiency^d^ | 5 (1.9) | 18 (8.5) | **<0.001** | 2 (1.6) | 3 (2.2) | 0.702 | 12 (11.1) | 6 (5.8) | 0.163 |
| Low ferritin ^e^ | 114 (43.5) | 46 (21.7) | **<0.001** | 54 (42.5) | 60 (44.4) | 0.753 | 26 (24.1) | 20 (19.2) | 0.392 |
| Folate Deficiency^f^ | 5 (1.9) | 3 (1.4) | 0.678 | 3 (2.4) | 2 (1.5) | 0.603 | 1 (0.9) | 2 (1.9) | 0.539 |
| Hypovitaminosis D^g^ | 97 (37.0) | 145 (68.4) | **<0.001** | 46 (36.2) | 51 (37.8) | 0.794 | 79 (73.1) | 66 (63.5) | 0.129 |
| **Change in Maternal Biochemical Profile in Third trimester from First trimester; Median (p25 to p75)** | | | | | | | | | |
| Serum B12 (pg/mL)^μ^ | 61.5 (-20.8 to 125.0) | 113.5 (12.5 to 176.5) | **<0.001** | 84.0 (12.0 to 145.0) | 48.0 (-37.0 to 106.0) | **0.004** | 136.5 (31.5 to 222.0) | 70.0 (6.3 to 136.0) | **0.001** |
| Homocysteine (μmol/L)^μ^ | -3.1 (-5.0 to -1.6) | -5.0 (-8.6 to -2.8) | **<0.001** | -3.2 (-5.2 to -1.7) | -3.0 (-4.8 to -1.4) | 0.283 | -5.3 (-9.5 to -3.3) | -4.5 (-7.9 to -2.4) | 0.112 |
| Holo Tc (pmol/L)^μ^ | 37.5 (18.2 to 58.9) | 42.5 (17.9 to 65.7) | 0.162 | 45.1 (21.5 to 66.7) | 32.5 (9.8 to 53.7) | **0.002** | 50.7 (22.1 to 82.4) | 35.1 (16.4 to 55.0) | **0.003** |
| Ferritin (ng/mL)^μ^ | -2.7 (-14.1 to 3.5) | -9.2 (-31.7 to 8.0) | 0.051 | -2.6 (-14.6 to 3.2) | -3.0 (-14.0 to 3.7) | 0.872 | -9.3 (-32.6 to 10.6) | -9.2 (-30.9 to 4.3) | 0.837 |
| Folate (ng/mL)^μ^ | 0.0 (0.0 to 1.1) | 0.1 (0.0 to 5.1) | **0.010** | 0.0 (0.0 to 2.1) | 0.0 (0.0 to 0.6) | 0.571 | 0.2 (0.0 to 5.0) | 0.0 (0.0 to 5.2) | 0.848 |
| Vitamin D (ng/mL)^μ^ | 6.5 (-1.2 to 13.0) | 3.8 (-0.3 to 9.1) | 0.098 | 6.7 (0.0 to 13.4) | 5.4 (-2.6 to 13.0) | 0.234 | 3.7 (-0.6 to 8.5) | 3.9 (0.0 to 9.7) | 0.458 |

*Values represent Mean (SD) or median (IQR) ^μ^  for continuous variables or Number (Percentage) for discrete variables; ANOVA test used for comparing groups for normally distributed continuous variables/Mann Whitney for non-normally distributed variable and Chi-Square test for discrete variables. Mean differences and 95% CI are provided only for normally distributed continuous variables.

^a^Anaemia: Hemoglobin <110g/L

^b^B12 Deficiency: Serum B12 <197pg/ml

^c^Hyperhomocystinemia: Serum Homocysteine ≥ 12μmol/L (with folate supplement), ≥ 15μmol/L (without folate supplement)

^d^Holo Tc Deficiency: Serum Holo TC <25.10pmol/L

^e^Low Ferritin: Ferritin <13ng/mL

^f^Folate Deficiency: Folate <4.4ng/mL

^g^Hypovitaminosis D: 25(OH)D <20ng/mL

**Supplementary Table S8: Sub-Group Analysis: Country-Wise and Group by Country comparison of Infant Outcomes**

|  | **India Overall (*n=*283)** | **Nepal**  **Overall (*n=*248)** | ***p-value*** | **INDIA  (*n=*283)** | | ***p-value*** | **NEPAL  (*n=*248)** | | ***p-value*** |
| --- | --- | --- | --- | --- | --- | --- | --- | --- | --- |
|  |  |  |  | **Group A (*n=*135)** | **Group B (*n=*148)** |  | **Group A (*n=*120)** | **Group B  (*n=*128)** |  |
| **Assessment at 9-12 months of age** | | | | | | | | | |
| Weight, grams | 9020.9±1023.5 | 8644.9±1082.7 | **<0.001** | 8985.8±994.7 | 9052.8±1051.4 | 0.584 | 8748.2±1054.5 | 8548.2±1103.8 | 0.146 |
| Weight for Age Z score | 0.3±0.9 | 0.04±0.9 | **0.004** | 0.2±0.9 | 0.4±1.0 | 0.188 | 0.2±0.9 | -0.1±1.0 | **0.070** |
| Length, cm | 72.6±3.0 | 70.8±2.9 | **<0.001** | 72.8±2.9 | 72.4±3.1 | 0.217 | 71.1±2.5 | 70.5±3.1 | 0.141 |
| Height for Age Z score | 0.4±1.2 | -0.1±1.1 | **<0.001** | 0.5±1.2 | 0.4±1.2 | 0.587 | 0.03±1.1 | -0.2±1.2 | 0.068 |
| Head circumference, cm | 45.0±1.5 | 44.2±1.7 | **<0.001** | 45.1±1.5 | 44.9±1.5 | 0.278 | 44.3±1.7 | 44.2±1.6 | 0.747 |
| Breast feeding at 3 months (*n (%)*) | 204(94.9) | 15(30.6) | **<0.001** | 94(94.0) | 110(95.6) | 0.583 | 5(20.8) | 10(40.0) | 0.146 |
| Breast feeding at 6 months (*n (%)*) | 196(88.3) | 5(33.3) | **<0.001** | 88(85.4) | 108(90.8) | 0.219 | 3(37.5) | 2(28.6) | 0.714 |
| DASII, Percentile, Motor (mean (SD)) | 35.0±27.5 | 63.4±27.3 | **<0.001** | 35.6±27.0 | 34.5±28.0 | 0.726 | 65.9±26.5 | 61.1±28.0 | 0.166 |
| DASII, Percentile, Mental (mean (SD)) | 76.0±31.5 | 71.5±36.1 | 0.125 | 81.5±27.1 | 71.0±34.3 | **0.005** | 74.0±35.6 | 69.1±36.4 | 0.281 |
| Bradley HOME Inventory: Total score | 35.4±4.1 | 35.2±2.9 | 0.727 | 35.6±4.2 | 35.1±4.1 | 0.279 | 35.1±2.6 | 35.4±3.1 | 0.578 |
| **Infant Biochemical Parameters:** **Median (P25 to P75) ^μ^** | | | | | | | | | |
| Haemoglobin (g/L) | 113.0 (106.5 to 120.0) | 115.0 (109.0 to 122.0) | 0.023 | 112.0 (106.5 to 118.0) | 114.0 (106.3 to 120.0) | 0.410 | 114.5 (108.0 to 122.3) | 115.0 (110.0 to 122.0) | 0.784 |
| **Infant Micronutrient Deficiencies, *n (%)*** | | | | | | | | | |
| Anaemia^a^ | 83 (33.5) | 64 (26.4) | 0.622 | 40 (33.9) | 43 (33.1) | 0.891 | 33 (28.2) | 31 (24.8) | 0.548 |
| Vitamin B12 Deficiency^b^ | 66 (27.0) | 68 (28.6) | 0.226 | 27 (23.3) | 39 (30.5) | 0.207 | 31 (27.2) | 37 (29.8) | 0.652 |
| Hyperhomocystinemia^c^ | 45 (18.4) | 84 (35.3) | 0.083 | 19 (16.4) | 26 (20.2) | 0.446 | 34 (29.8) | 50 (40.3) | 0.090 |
| Holo Tc Deficiency^d^ | 23 (9.4) | 45 (18.9) | 0.407 | 9 (7.9) | 14 (10.8) | 0.443 | 20 (17.5) | 25 (20.2) | 0.606 |

*Values represent Mean (SD) or median (IQR)^μ^  for continuous variables or Number (Percentage) for discrete variables; ANOVA test used for comparing groups for normally distributed continuous variables/Mann Whitney for non-normally distributed variable and Chi-Square test for discrete variables. Mean differences and 95% CI are provided only for normally distributed continuous variables.

^a^Anaemia: Hemoglobin <110g/L

^b^B12 Deficiency: Serum B12 <259pg/ml

^c^Hyperhomocystinemia: Serum Homocysteine ≥ 8μmol/L (with folate supplement), 1 ≥ 0 μmol/L (without folate supplement)

^d^Holo Tc Deficiency: Serum Holo Tc <25.10pmol/L

**Supplementary Table S9A. Prespecified subgroup analyses for Mental DQ (within-subgroup comparisons)**

| **Subgroup stratum** | **n (Group A)** | **Group A mean** ± **SD** | **n (Group B)** | **Group B mean** ± **SD** | **Mean difference (A–B)** | **p-value** |
| --- | --- | --- | --- | --- | --- | --- |
| Maternal baseline B12 status: Deficient | 137 | 103.7 ± 8.3) | 141 | 101.8 ± 8.6 | 1.90 | 0.061 |
| Maternal baseline B12 status: Non-deficient | 118 | 103.7 ± 7.0 | 135 | 101.8 ± 9.1 | 1.90 | 0.059 |
| APGAR at 1 min: <7 | 49 | 102.8 ± 8.9 | 41 | 102.5 ± 9.5 | 0.30 | 0.864 |
| APGAR at 1 min: ≥7 | 206 | 104.0 ± 7.4 | 235 | 101.7 ± 8.8 | 2.30 | **0.004** |
| Birth size: SGA | 76 | 102.9 ± 8.1 | 91 | 101.6 ± 8.6 | 1.30 | 0.329 |
| Birth size: Non-SGA | 179 | 104.1 ± 7.6 | 185 | 101.9 ± 9.0 | 2.20 | **0.012** |
| Risk status: High-risk | 136 | 102.9 ± 8.2 | 145 | 101.7 ± 8.9 | 1.20 | 0.259 |
| Risk status: Low-risk | 119 | 104.7 ± 7.1 | 131 | 101.9 ± 8.9 | 2.80 | **0.006** |

**Supplementary Table S9B. Prespecified subgroup analyses for Motor DQ (within-subgroup comparisons)**

| **Subgroup stratum** | **n (Group A)** | **Group A mean** ± **SD** | **n (Group B)** | **Group B mean** ± **SD** | **Mean difference (A–B)** | **p-value** |
| --- | --- | --- | --- | --- | --- | --- |
| Maternal baseline B12 status: Deficient^ | 137 | 98.4 ± 9.8 | 141 | 98.1 ± 11.5 | 0.30 | 0.807 |
| Maternal baseline B12 status: Non-deficient^ | 118 | 98.3 ± 10.2 | 135 | 96.6 ± 9.8 | 1.70 | 0.187 |
| APGAR at 1 min: <7 | 49 | 102.8 ± 11.1 | 41 | 101.6 ± 9.4 | 1.20 | 0.588 |
| APGAR at 1 min: ≥7 | 206 | 97.3 ± 9.4 | 235 | 96.6 ± 10.8 | 0.70 | 0.496 |
| Birth size: SGA | 76 | 97.9 ± 8.4 | 91 | 97.8 ± 11.3 | 0.10 | 0.985 |
| Birth size: Non-SGA | 179 | 98.6 ± 10.5 | 185 | 97.2 ± 10.4 | 1.40 | 0.200 |
| Risk status: High-risk* | 136 | 99.3 ± 9.8 | 145 | 98.4 ± 11.0 | 0.90 | 0.453 |
| Risk status: Low-risk* | 119 | 97.3 ± 10.0 | 131 | 96.3 ± 10.3 | 1.00 | 0.442 |

^ Maternal B12 levels < 197 pg/ml were defined as deficient

Note: SGA is defined as Small for Gestational Age (<10^th^ centile by WHO charts^26^)

* High risk mother and infant were defined as: Maternal morbidity during pregnancy (including gestational diabetes, pregnancy-induced hypertension/pre-eclampsia/eclampsia and absent or reversed end diastolic flow, Birth weight z-score categorized as high-risk (small for gestational age [SGA]: <10th centile for birth weight), Gestation categorized as very preterm (28-32 weeks gestation), Higher risk neonatal course categorized as high risk (if neonate has: APGAR score <7 at 1 minute, hypoglycemia, hyperbilirubinemia or prolonged neonatal intensive care stay >7 days), Infants with documented neurological disease/anomaly/illness
