# Supplementary material for: Maternal supplementation of vitamin B12 in predominantly vegetarian pregnant women improves their vitamin B12 status and the neurodevelopment of their infants: the MATCOBIND multicentric double-blind randomised control trial
Source: BMJ Paediatr Open. 2026 Mar 18;10(1):e004112. doi: 10.1136/bmjpo-2025-004112 (PMC13034390; doi:10.1136/bmjpo-2025-004112)
Supplement: Supplementary file 2 [file bmjpo-2025-004112.reviewer_comments.pdf]

PEER REVIEW HISTORY

BMJ Paediatrics Open publishes all reviews undertaken for accepted manuscripts. Reviewers are asked to complete a checklist review form and are provided with free text boxes to elaborate on their assessment. These free text comments are reproduced below.

ARTICLE DETAILS

**TITLE (PROVISIONAL)**	Maternal supplementation of vitamin B12 in predominantly vegetarian pregnant women improves their vitamin B12 status and the neurodevelopment of their infants: The MATCOBIND multi-centric double-blind randomised control trial
**AUTHORS**	Nagpal, Jitender<br>Mathur, Manu<br>Rawat, Swapnil<br>Singhal, Atul<br>Pant, Rajendra<br>Shah, Anita<br>Nixon, Laura<br>Mishra, Vijay K<br>Nagrath, Deepti<br>Heys, Michelle<br>Cortina-Borja, Mario<br>Michie, Colin<br>Gautam, Jageshwor<br>Karmacharya, Shailendra Bir<br>Rai, Snighda<br>Lakhanpaul, Monica

VERSION 1 – REVIEW

**REVIEWER NAME**	Kvestad, Ingrid
**REVIEWER AFFILIATION**	NORCE Norwegian Research Centre AS
**REVIEWER CONFLICT OF INTEREST**	No Conflict of Interest
**AI DISCLOSURE**	No, I have not used AI
**DATE REVIEW RETURNED**	15-Oct-2025

**GENERAL COMMENTS**	Thank you for letting me review the report from this important study. I find the study interesting and study findings important to publish. The paper draft has several overall weaknesses, however. Overall, there is a need for polishing, there are inconsistencies in the use of punctuations, use of wrong references, abbreviations that lacking definition (e.g. NCR) etc. I have also several specific comments below.<br><br>Abstract:<br>- Baseline differences should not be tested for significance (this is also according to CONSORT list).<br>- Reporting the results;<br>o what is the p=0.008 referring to for the mental development quotient? Difference between groups in the DQ? What is then the p=0.007 referring to?<br>o On the increase of B12 status – what are the numbers in parenthesis? Difference from 1st to 3rd semester or absolute value at 3rd trimester?? What is the p-value (p<0.001) referring to? The

	difference in increase? What is the corresponding effect size? In general, the increase is more interesting than the simple p-value (for Holotranscobalamin).<br>- In the conclusion – "infant mental DQ" does not really make sense in a developmental perspective. Consider writing out? What is the Mental Development quotient measuring?<br><br>Summary boxes:<br>Revisit for stringency and consistency.<br><br>Introduction:<br>Everything is perhaps there, but I find the flow to be poor. And the links between the different parts to be low. Also, there are some mistakes in the discussion of previous studies and findings. Please review thoroughly, also review the references.<br><br>For instance:<br>- Line 12 – the references here are two trials from Nepal, one on infant supplementation and one on maternal supplementation, both revealing null findings. These are not accurate for the sentence. There are small trials from high-income countries showing an effect of B12 supplementation in infants, so the content on the sentence in line 12 is incorrect. I also see that reference 16 and 21 are the same.<br>- I find there is a missing link over from the literature to your rationale (starting in line 29). Why are you doing this trial? Why in vegetarian mothers only? Why do you conduct a study in mothers with particularly low status? What are the benefits of your approach compared to previous ones? Why different doses? Why not a full placebo? I think these points should be discussed, before you describe briefly your own trial.<br>- And moreover: What are the countries? You say "both countries", still these have not been mentioned yet. It is also confusing that you describe the enrolled mothers here (on SES and nutritional status). The flow is particularly poor in the last paragraph of the introduction.<br><br>Methods:<br>Please make sure to use the CONSORT list in reporting a clinical trial – all element should be included.<br><br>- I would like more information on the study design and study setting, it is not merely sufficient to refer to the protocol paper.<br><br>- On patient public involvement the paragraph says nothing on what was done, merely on good intentions.<br><br>- Please revisit the definition of predominantly vegetarian paragraph – poor sentences should be reworded.<br><br>- Randomisation and Masking:<br><br>o who did the randomization?<br>o sentences on line 30-32 says the same.<br><br>- Study outcomes:<br>o What norms were used to convert into composite scores? Are there local norms?<br>o B12 status in the mother and child were key secondary outcomes?

	What about all the other outcomes reported in the tables? What are these?<br><br>- Trial protocol modifications – perhaps briefly state the modifications overall?<br><br>- Sample size considerations<br><br>o Reference 30 is incomplete<br>o What is the reference for 2 points being a clinically significant effect size? I find this to be quite small.<br><br>- Biochemical analyses:<br>o What are the references for biochemical deficiencies. There would be great variations in the field. I.e. I have never seen 197pg/ml used as a cut of for serum B12 (of note – is this cobalamin?), hardly ever 12 and 15 for homocysteine and 4.4 for folate.<br><br>- Data analysis:<br>o how was the WAZ-scores calculated?<br>o Why predefined adjustment variables to calculate adjusted outcomes in a RCT design?<br><br>Anything on ethics??<br><br><br><br>Results<br>I find the presentation of results quite confusing! Both in text and in tables. You should consider closely which results should be in main paper and what is supplementary. I think many of the results could be moved to supplementary in order to put forward the important findings.<br>- I question the level of details summarizing figure 1 (lines 23-32 side 10 and 1-3 side 11). I would draw out the essential (perhaps the last sentences of the paragraph?).<br>- Table 1 – significance testing does not make sense comparing a randomized sample. Please revise table. Text is ok.<br>- Table 2a. vitamin B12 status is a predefined secondary outcome. There is a lot of information on the other changes in micronutrient – both in table and detailed in text. I would consider moving these not relevant to vitamin status/OCM to supplementary and not report in such detail text. This would put forward your most relevant results.<br>- Table3a – is this background information that could be in supplementary and just briefly mentioned in text?<br>- Table 4<br>o You used age-corrected scores?<br>o How did you calculate the centiles? I cannot find the description in the Data analysis part.<br>o The first paragraph of page 12 is very confusing mixing the description of outcomes (development and growth) and other variables (breast-feeding, HOME), and biochemical information.<br>- Table 5: Why were these analyses performed? How do they inform the results? Why is it necessary to show adjusted estimates in a RCT?<br>- Table 6 and 7 – supplementary information?<br>o Are there anything called “shorter socio-economic status”? Perhaps lower?<br>- Perhaps reword: Results from subgroup analyses are shown in supplementary tables x to x. And then summarise the results. It is

	interesting (and should be put forward) that the difference is only seen in the Indian sample.<br><br>Table 1 – I would now show the overall numbers only per group. Mean diff and 95%CI and p-value do not belong in a baseline table (please see CONSORT checklist).<br><br>Table 2: should indicate clearly whether numbers are presented as mean (SD) or median(IQR).<br>The change estimates – what are these? How do they correspond with the 1st and 3rd<br>trimester measures? Third trimester Serum B12 concentration is not that different between groups – why is there such a large difference in change?<br><br>Tables 3a and b – I suggest move to supplementary – this is information that is only important as background information.<br><br>Table 4: How can breast feeding be an outcome? Is it related to maternal supplementation? It is important as background – but cannot be an outcome. HOME is the same.<br><br>Table 5: I do not understand these analysis.<br><br>Supplementary tables on subgroups – are there any patterns of which group that reveal a difference on the primary outcomes? Could these analysis (subgroup analysis on primary outcome) be presented on its own?<br><br>Discussion:<br>Are the biochemically differences seen at 3rd trimester clinically meaningful? A difference in tHcy of 5.3 and 5.7 – although significant – is it an important finding clinically?<br><br>Comparing with Chandyo et al – it is worth noting that the current trial did not observe a difference between group when analysis was restricted to the Nepali population?

VERSION 1 – AUTHOR RESPONSE

Introduction:
Everything is perhaps there, but I find the flow to be poor. And the links between the different parts to be low. Also, there are some mistakes in the discussion of previous studies and findings. Please review thoroughly, also review the references.

For instance:

Comment 1: Line 12 – the references here are two trials from Nepal, one on infant supplementation and one on maternal supplementation, both revealing null findings. These are not accurate for the sentence. There are small trials from high-income countries showing an effect of B12 supplementation in infants, so the content on the sentence in line 12 is incorrect. I also see that reference 16 and 21 are the same.

Response: We thank the reviewer for highlighting this important issue. We agree that our original sentence was overly broad and did not adequately reflect the heterogeneity of the infant supplementation literature. We have revised the text to specify that the large infant supplementation trial(s) in Nepal did not demonstrate neurodevelopmental benefit, while acknowledging that small, randomized trials from high-income settings (e.g., Norway) have reported improvements in infant motor

outcomes and/or symptoms after cobalamin supplementation, particularly among infants with biochemical evidence of impaired cobalamin function (P5, L13-23). We have updated the wording accordingly and added/ revised the relevant references.

Comment 2: I find there is a missing link over from the literature to your rationale (starting in line 29). Why are you doing this trial? Why in vegetarian mothers only? Why do you conduct a study in mothers with particularly low status? What are the benefits of your approach compared to previous ones? Why different doses? Why not a full placebo? I think these points should be discussed, before you describe briefly your own trial.
Response: We thank the reviewer for their thoughtful insight. In order to enhance clarity, we have now significantly revised the introduction lines before eliciting the aims and objectives to reflect and respond to the reviewer's questions. (P6, L1-21).

Comment 3: And moreover: What are the countries? You say "both countries", still these have not been mentioned yet. It is also confusing that you describe the enrolled mothers here (on SES and nutritional status). The flow is particularly poor in the last paragraph of the introduction.

Response: We thank the reviewer for this helpful feedback. We agree that the final paragraph of the Introduction had poor flow and used the phrase "both countries" before specifying the countries, while also mixing background policy information and participant characteristics. We have therefore rewritten the last part of the Introduction to (i) clearly name India and Nepal and the two study settings, (ii) provide a more coherent rationale for focusing on predominantly vegetarian women (high risk of low dietary B12), and (iii) succinctly justify the dose comparison (250 μg vs 50 μg) including the ethical rationale for not using a full placebo. We have also removed the confusing description of enrolling women based on socioeconomic/nutritional status and retained only the relevant eligibility details (P6, L1-21).

Methods

Comment 1: Please make sure to use the CONSORT list in reporting a clinical trial – all element should be included. I would like more information on the study design and study setting, it is not merely sufficient to refer to the protocol paper. On patient public involvement the paragraph says nothing on what was done, merely on good intentions.
Response: As recommended, we have included additional information in the methodology section, specifying the study design, setting, masking, randomization and PPI work.

Comment 2: Please revisit the definition of predominantly vegetarian paragraph – poor sentences should be reworded.
Response: The paragraph the definition of predominantly vegetarian has been modified as suggested (P8, L20-23).

Comment 3: Randomization and Masking: who did the randomization? sentences on line 30-32 says the same.
Response: The suggestions had been added in the revised version of the manuscript (P8, L27-28).

Study outcomes

Comment 1: What norms were used to convert into composite scores? Are there local norms?
Response: The Developmental Assessment Scale for Indian Infants (DASII) uses standardized norms derived from its original validation on Indian infants. Raw scores obtained from the Mental and Motor scales are first converted into age-equivalent scores based on item placement within the scale.

These age-equivalent scores are then used to calculate the Mental Age (MA) and Motor Age (MoA) for each child. Composite scores are expressed as Developmental Quotients (DQ), calculated using the formula:

$$DQ = \frac{\text{MA or MoA}}{\text{Chronological Age}} \times 100$$

Thus, the Mental Developmental Quotient (MeDQ) and Motor Developmental Quotient (MoDQ) represent standardized scores adjusted for chronological age.

Regarding local norms:

Yes, DASII norms are based on local population data (referred to as Baroda norms) and were specifically developed and standardized. These norms account for cultural and developmental variations relevant to the local context.

Ref: Misra N, Pathak P. Developmental assessment scales for Indian infants (DASII): Manual. *Baroda, MS University of Baroda* 1996

Comment 2: B12 status in the mother and child were key secondary outcomes? What about all the other outcomes reported in the tables? What are these?
Response: Thank you for this comment. We agree that the outcomes needed to be presented more clearly and that several variables reported in the tables/text could be misinterpreted as outcomes. In the revised manuscript, we have explicitly restructured the outcomes section to distinguish secondary and exploratory outcomes from explanatory/background variables**.**

As presented in the revised version (P11, 1-8), the outcomes are now specified as follows:

Secondary outcomes

- Change in B-12 status (vitamin B-12 level, Homocysteine, and Holo-transcobalamin) of mother between first (<12 weeks' gestation) and third trimester (> 27 weeks' gestation);
- B-12 status (vitamin B-12 level, Homocysteine, and Holo-transcobalamin) of infants at 9 months (±2 weeks) post-partum.

Exploratory outcomes

- Haemoglobin (Hb) of infants at 9 months (+14 days) post-partum;
- Infant anthropometry (weight, length/height, and head circumference) at 9 months after birth in all subjects.

We further clarify that folate, vitamin D, and ferritin were not trial outcomes**;** these were collected and reported as explanatory variables to describe the broader micronutrient context, assess coexisting deficiencies, and support interpretation of the neurodevelopmental findings (given that multiple micronutrient deficiencies may coexist and potentially influence growth and development). We have accordingly revised the text and table labelling to avoid any confusion regarding these measures being outcomes.

Comment 3: Trial protocol modifications – perhaps briefly state the modifications overall?
Response: Thank you for this comment. We have now included a brief summary of the major trial protocol modifications in the revised manuscript (P11, L10-16).

Sample size considerations

Comment 1: Reference 30 is incomplete
Response: The reference has been modified as suggested.

Comment 2: What is the reference for 2 points being a clinically significant effect size? I find this to be quite small.
Response: We identified 2 DQ points (~0.28 SD) as the minimum clinically important difference, referencing distribution-based on Minimal Clinically Important Difference (MCID) conventions (where ≥0.2 SD is generally accepted as meaningful—equivalent to 1.5 DQ points). Typically, distribution-based methods consider changes within 0.2–0.5 SD to be significant in clinical measurement, especially when dealing with continuous standardized outcomes. This method is widely applied across health outcomes studies to highlight effects that are practically important, not just statistically significant, and to inform trial power calculations. This threshold was established in our protocol paper and informed our sample size justification. Further, According to DASII norms (mean 100, SD 15), a 2-point increase would lower the proportion scoring below 85 from 15.9% to 12.9% (a reduction of 3.0 percentage points). Such a shift can be quite impactful in resource-limited environments like LMICs. These findings show that improving by 2 points effectively reduces the proportion of at-risk cases, aligning with DASII's role as a key diagnostic tool for infant development in India and supporting the MCID approach.

- Jaeschke R, Singer J, Guyatt GH. *Measurement of health status: Ascertaining the minimal clinically important difference.* Control Clin Trials. 1989;10(4):407–415. (PMID: 2691207)
- Madaan P, Saini L, Sondhi V. *Development Assessment Scale for Indian Infants: A Systematic Review and Perspective on Dwindling Cutoffs.* Indian J Pediatr. 2021;88(9):918–920. (PMID: 33555568)

Biochemical analyses:

Comment 1: What are the references for biochemical deficiencies. There would be great variations in the field. i.e. I have never seen 197pg/ml used as a cut of for serum B12 (of note – is this cobalamin?), hardly ever 12 and 15 for homocysteine and 4.4 for folate.

Response: Thank you for raising this important point regarding the biochemical cut-offs. The thresholds used in our study (serum vitamin B12 <197 pg/mL, homocysteine ≥12 µmol/L with folate and ≥15 µmol/L without folate, and serum folate <4.4 ng/mL) were those specified by the NABL-certified reference laboratory that processed all samples. These values reflect the assay-specific reference ranges validated for clinical interpretation in pregnancy.

While laboratory-specific cut-offs can vary, the ranges we used are consistent with those cited in the literature and international guidelines:

- **Vitamin B12:** Vitamin B12 deficiency at defined at <148 pmol/L (~200 pg/mL) and marginal status up to 221 pmol/L (~300 pg/mL)
- **Homocysteine:** Functional hyperhomocysteinemia linked to B12/folate deficiency is commonly defined as ≥12–15 µmol/L
- **Folate:** Maternal studies in India use thresholds around 4–5 ng/mL for deficiency

Reference: Refsum H, Yajnik CS, Gadkari M, Schneede J, Vollset SE, Örning L, Guttormsen AB, Joglekar A, Sayyad MG, Ulvik A, Ueland PM. Hyperhomocysteinemia and elevated methylmalonic acid indicate a high prevalence of cobalamin deficiency in Asian Indians. The American journal of clinical nutrition. 2001 Aug 1;74(2):233-41.

We have clarified in the revised manuscript that these cut-offs were laboratory-defined but align with internationally recognized ranges for deficiency and marginal status (P12, L8-9).

Data analysis

Comment 1: How was the WAZ-scores calculated?
Response: WAZ (weight-for-age z-score) score was calculated using WHO Child Growth Standards using an LMS method: the child's weight is standardized relative to sex- and age-specific L, M, and S reference values, a specific package (module) named- zscore06 was used to calculate the WAZ in Stata software (version 14.0)

Reference- Leroy, Jef L (2011). zscore06: Stata command for the calculation of anthropometric z-scores using the 2006 WHO child growth standards
https://ideas.repec.org/c/boc/bocode/s457279.html#download

Comment 2: Why predefined adjustment variables to calculate adjusted outcomes in a RCT design?
Response: Predefined adjustment variables were used in RCT to calculate adjusted outcomes by accounting for chance baseline differences covariates that might strongly influence the primary outcome, thereby increasing statistical precision and power without introducing bias. This technique reduces variability in outcome estimates beyond what randomization alone achieves. This is further detailed in response to Comment 7 under Results

Comment 3: Anything on ethics??
Response: We have included a detailed statement in the Methods section explaining the ethical approvals sought to carry out this study (P 12, L 32-34 & P 13, L1-5).

Results

Comment 1: I find the presentation of results quite confusing! Both in text and in tables. You should consider closely which results should be in main paper and what is supplementary. I think many of the results could be moved to supplementary in order to put forward the important findings.
Response: We appreciate the reviewer's feedback regarding the presentation of results. We have thoroughly reviewed and reorganized the Results section, limiting ourselves to the main findings as recommended. Less critical details have been moved to the supplementary material to ensure that the primary results are clearly highlighted in the main paper.

Comment 2: I question the level of details summarizing figure 1 (lines 23-32 side 10 and 1-3 side 11). I would draw out the essential (perhaps the last sentences of the paragraph?).
Response: We thank the reviewer for highlighting this and have condensed the description of Figure 1 in the Results section. The detailed step-by-step account of participant screening and exclusions has been removed, and we now provide only a concise summary of the participant flow in place of the original description (P13, L7-11).

Comment 3: Table 1 – significance testing does not make sense comparing a randomized sample. Please revise table. Text is ok.
Response: We agree with the observation of the reviewer and have removed significance testing in Table 1.

Comment 4: Table 2a. vitamin B12 status is a predefined secondary outcome. There is a lot of information on the other changes in micronutrient – both in table and detailed in text. I would consider moving these not relevant to vitamin status/OCM to supplementary and not report in such detail text. This would put forward your most relevant results.

Response: Thank you for this suggestion. We agree that the predefined secondary outcome was maternal vitamin B12 status and that presenting extensive details on other micronutrients in the text may detract from the main focus of the manuscript. These additional micronutrient data (iron, folate, vitamin D, ferritin) were collected as part of the protocol to describe the overall nutritional profile and explore potential confounders, but they are not central to the hypothesis being tested.

In line with your recommendation, we have streamlined the Results section by removing detailed narrative descriptions of non-B12 micronutrient changes from the text while retaining the complete data in Table 3 for transparency and reader reference. This approach ensures that the main text emphasises the most relevant findings—maternal B12 status and infant neurodevelopment—while still providing full data in the tables for those interested in the broader nutritional context.

Comment 5: Table3a – is this background information that could be in supplementary and just briefly mentioned in text?
Response: The table has been moved to supplementary document (Table S3).

Comment 6: Table 4
(i) You used age-corrected scores?

Response: Thank you for this question. We did not apply any additional post-hoc “age correction” to the neurodevelopment scores. The primary outcome was the DASII Developmental Quotient (DQ), which is inherently age-standardised because it is calculated as *developmental age / (chronological age) × 100* (separately for Mental and Motor domains). Therefore, the DQ already accounts for the child’s age at the time of assessment. For preterm infants, assessments were conducted and interpreted using corrected age (corrected gestational age), consistent with standard developmental practice and as specified in the study procedures. For term infants, chronological age at assessment was used. No other age-adjustment was performed beyond these standard procedures. The same has been clarified in footnote to Table 2.

(ii) How did you calculate the centiles? I cannot find the description in the Data analysis part.
Response: Thank you for your query regarding the calculation of centiles (percentile ranks) for DASII scores. The centiles in our analysis were calculated strictly according to the procedure described in the DASII manual (Developmental Assessment Scales for Indian Infants, Manual, Baroda, MS University of Baroda). As outlined in the manual, after obtaining the raw scores for the Mental and Motor scales, these are first converted to age-equivalent scores (Mental Age and Motor Age). The Developmental Quotients (DQ) are then calculated using the following formulas:

- **Mental DQ (DMeQ) = Mental Age / Chronological Age × 100**
- **Motor DQ (DMoQ) = Motor Age / Chronological Age × 100**

To derive the centile (percentile rank), the child’s DQ score is referenced against the normative tables provided in the DASII manual (Appendix IV). Specifically, the manual states that the column corresponding to the child’s age group and the row of the child’s total score are used to obtain the percentile rank from the normative tables. This method ensures that each child’s DQ is converted into a percentile rank appropriate for their age group, reflecting their position relative to the normative sample. The clinical interpretation and use of these percentile ranks is further detailed in Appendix VII of the DASII manual.

Reference: Misra N, Pathak P. Developmental assessment scales for Indian infants (DASII): Manual. Baroda, MS University of Baroda 1996

(iii) The first paragraph of page 12 is very confusing mixing the description of outcomes (development and growth) and other variables (breast-feeding, HOME), and biochemical information.
Response: Thank you for pointing this out. We have rewritten and restructured the first and second paragraph on page 12 to clearly separate the primary infant outcomes from the other variables.
The first sentence on page 12 of previous version now focuses on the infant neurodevelopment and growth outcomes. We clearly state the primary result that infants in the 250 µg B12 group had higher Mental Developmental Quotient (DQ) scores than those in the 50 µg group, while noting that Motor DQ and growth (anthropometric measurements) did not significantly differ between groups (P13, L24-32).

Comment 7: Table 5: Why were these analyses performed? How do they inform the results? Why is it necessary to show adjusted estimates in a RCT?

Response: Thank you for your comment regarding the inclusion of adjusted analyses in Table 5. Although randomization in RCTs is designed to balance both known and unknown confounders, chance imbalances in important prognostic variables can still occur, particularly in multi-center studies or when sample sizes are moderate. In our trial, we pre-specified (documented in protocol paper) adjustment for key covariates (study site, infant gender, breastfeeding status at 3 months, weight-for-age z-score at birth, change in WAZ from birth to 3 months, and maternal BMI at recruitment) because these factors are well-established predictors of neurodevelopmental outcomes and could influence the primary endpoint independent of the intervention. Presenting adjusted analyses increases the precision and robustness of our findings, ensuring that the observed effect of vitamin B12 supplementation is not confounded by baseline differences or site-specific factors. Notably, the adjusted difference in Mental DQ was higher than the unadjusted difference, highlighting the importance of accounting for these covariates. This approach is consistent with CONSORT guidelines, which recommend reporting both unadjusted and adjusted estimates, especially when covariate adjustment is pre-specified or when important prognostic factors are known. Thus, the adjusted analyses in Table 5 provide a more reliable and interpretable estimate of the intervention effect in our RCT, strengthening the validity of our conclusions

Comment 8: Table 6 and 7 – supplementary information?
Response: Thank you for your suggestion regarding Tables 6 and 7. We agree that these tables contain supplementary information and have moved them to the supplementary material as recommended (Table S7 and S8).

Comment 9: Are there anything called “shorter socio-economic status”? Perhaps lower?
Response: The line “shorter socio-economic status” has been modified in the revised manuscript (P15, L10).

Comment 10: Perhaps reword: Results from subgroup analyses are shown in supplementary tables x to x. And then summarise the results. It is interesting (and should be put forward) that the difference is only seen in the Indian sample.
Response: Thank you for this helpful suggestion. We agree that the subgroup analyses should be clearly signposted and summarised in the main text. Accordingly, we have reworded the Results section to direct readers to the subgroup tables and added a concise summary of the key finding. In the revised tables, country-stratified results are presented explicitly.

Tables

Comment 1: Table 1 – I would now show the overall numbers only per group. Mean diff and 95%CI and p-value do not belong in a baseline table (please see CONSORT checklist).
Response: We have revised the Table 1 as suggested.

Comment 2: Table 2: should indicate clearly whether numbers are presented as mean (SD) or median (IQR).
The change estimates – what are these? How do they correspond with the 1st and 3rd trimester measures? Third trimester Serum B12 concentration is not that different between groups – why is there such a large difference in change?

Response:

1. **Clarification of summary statistics:**
   We agree that the table should clearly indicate whether values are presented as *mean (SD)* or *median (IQR)*. These are added to the table footnotes for transparency.
2. **Explanation of "change estimates":**
   The change estimates represent the difference between third-trimester and first-trimester values for each participant, summarized as median (IQR). This was pre-specified in the protocol to capture the magnitude of improvement during supplementation.

3. **Why is the change in serum B12 large despite modest third-trimester differences?**
   The apparent discrepancy arises from two factors:
   - **Baseline variation among those with paired data:** While first-trimester values for the full cohort (n=704) were similar across groups, the subset with paired measurements (n=474) had slightly lower baseline B12 in Group A compared to Group B.
   - **Combined effect of baseline gap and third-trimester difference:** When calculating change, the lower starting point in Group A combined with a ~30 pg/mL higher third-trimester level amplifies the net improvement. Thus, the change metric reflects both the intervention effect and the baseline distribution of the paired sample.

We have clarified these points in the revised manuscript and explicitly note the difference in sample sizes (first trimester n=704 vs third trimester n=474) in the table footnote to avoid misinterpretation.

Comment 3: Tables 3a and b – I suggest move to supplementary – this is information that is only important as background information.
Response: Table 3a and b has been moved to supplementary tables (Table S3).

Comment 4: Table 4: How can breast feeding be an outcome? Is it related to maternal supplementation? It is important as background – but cannot be an outcome. HOME is the same.

Response: Thank you for this observation. We agree that breastfeeding status and HOME inventory scores are not trial outcomes but rather contextual variables that describe the infant's environment and care practices. These were originally included in Table 4 as part of the chronological sequence of infant assessments but were never intended to be interpreted as outcomes of maternal supplementation.
In response to your suggestion, we have removed breastfeeding and HOME scores from the table of outcomes and revised the Results section (P13, L31) to present them as explanatory/background variables rather than outcomes (Table 2).

Comment 5: Table 5: I do not understand this analysis.
Response: Addressed in the result section under Comment 7.

Comment 6: Supplementary tables on subgroups – are there any patterns of which group that reveal a difference on the primary outcomes? Could this analysis (subgroup analysis on primary outcome) be

presented on its own?
Response: Thank you for your suggestion regarding subgroup analyses. These analyses were pre-specified in the protocol to explore potential effect modification on the primary outcome. While we have retained the subgroup analyses for the revised version for transparency, we are seeking to balance as a detailed discussion of each subgroup is beyond the scope of this paper and could detract from the main objectives. We have summarized the key patterns (e.g., greater benefit in Indian participants and low-risk pregnancies) in the main text and all subgroup analyses other than India vs Nepal is now briefly presented in Supplementary Table S6-S9. If acceptable, we would prefer to reserve some of the more granular subgroup analyses for a separate secondary analyses paper focused specifically on effect modification and contextual factors, where they can be discussed in depth.

Discussion

Comment 1: Are the biochemically differences seen at 3rd trimester clinically meaningful? A difference in tHcy of 5.3 and 5.7 – although significant – is it an important finding clinically?

Response: Thank you for your question regarding the clinical significance of the difference in total homocysteine (tHcy) levels observed at the third trimester (5.3 vs 5.7 μmol/L). We agree that, although this difference reached statistical significance ($p = 0.036$), this small absolute difference may not be clinically meaningful. Our discussion and conclusions focus on the improvement in vitamin B12 status and neurodevelopmental outcomes, rather than on differences in tHcy. We have clarified this point further in the revised manuscript to avoid any potential over-interpretation of this finding (P15, L26-27).

Comment 2: Comparing with Chandyo et al – it is worth noting that the current trial did not observe a difference between group when analysis was restricted to the Nepali population?
Response: Thank you for this insightful suggestion. We have now inserted a separate discussion point highlighting this observation (P16, L30-34 and P17, L1-7).

VERSION 2 – REVIEW

**REVIEWER NAME**	Kvestad, Ingrid
**REVIEWER AFFILIATION**	NORCE Norwegian Research Centre AS
**REVIEWER CONFLICT OF INTEREST**	No Conflict of Interest
**AI DISCLOSURE**	No, I have not used AI
**DATE REVIEW RETURNED**	17-Feb-2026

**GENERAL COMMENTS**	My queries have been taken care of and the manuscript is improved. I do suggest a final change in the abstract to include in the conclusion the important fact that this finding is in vegetarian mothers.
